# Tissue microarray profiling and integrative proteomics indicate the modulatory potential of *Maytenus royleanus* in inhibition of overexpressed TPD52 in prostate cancers

**DOI:** 10.1038/s41598-021-91408-8

**Published:** 2021-06-07

**Authors:** Maria Shabbir, Hasan Mukhtar, Deeba Syed, Suhail Razak, Tayyaba Afsar, Ali Almajwal, Yasmin Badshah, Dara Aldisi

**Affiliations:** 1 Atta-ur-Rahman School of Applied Biosciences, National University of Sciences and Technology, Islamabad, Pakistan; 2 Department of Dermatology, University of Wisconsin, Madison, USA; 3 Department of Community Health Sciences, College of Applied Medical Sciences, King Saud University, KSA, Riyadh, Saudi Arabia

**Keywords:** Biochemistry, Cancer

## Abstract

*Maytenus roylanus* (MEM) is a plant with anti-proliferative effects against prostate cancer. We aimed to explore the mechanism of action of MEM in prostate cancer (PCa) by employing an in vitro global proteome approach to get useful information of various signaling pathways and effected genes to define the mechanism of MEM action in prostate cancer. We conducted a global proteome analysis of CWR22Rv1after treatment with methanolic extract of MEM. The result of the proteomic profiling of in vitro PCa cells demonstrated the reduction in tumor protein D52 (TPD52) expression after treatment with methanolic extract of MEM. Down-regulation of TPD52 expression at mRNA level was observed by MEM treatment in CWR22Rν1 and C_4-2_ cells in a dose-dependent fashion probably by cleavage of Caspase 3 and PARP, or by modulation of cyclin-dependent kinases in CWR22Rν1 and C_4-2_ cells. The progressive character of the TRAMP model demonstrates a chance to evaluate the potential of chemo-preventive agents for both initial and late stages of prostate cancer development, and induction in TPD52 protein expression with development as well as the progression of prostate cancer was observed in the TRAMP model. Analyses of the tissue microarray collection of 25 specimens confirmed the clinical significance of our findings identifying TPD52 as a potential marker for PCa progression. We determined that knockdown of TPD52 (CWR22Rν1 cells), a considerable downregulation was seen at the protein level. Downregulation of TPD52 inhibited the migration and invasive behavior of prostate cancer cells as observed. Moreover, we observed that the siRNA-TPD52 transfection of CWR22Rν1 cells resulted in tumor growth inhibition with a marked reduction in the secretion of prostate-specific antigen (PSA) in the serum. Intraperitoneal injection of MEM considerably slowed tumor growth in athymic mice, inhibited TPD52 expression, and caused a marked reduction in PSA levels of serum as demonstrated by immunoblot screening and immune-histochemical staining. This report illustrates a molecular overview of pathological processes in PCa, indicating possible new disease biomarkers and therapeutic targets.

## Introduction

Prostate cancer is the second common malignancy (after lung cancer) in men all-inclusive, counting 1,276,106 new cases and causing 358,989 deaths (3.8% of all deaths caused by cancer in men) in 2018^1, 2^. The occurrence and death rate of prostate cancer all-inclusive correlate with cumulative age with the average age at the time of diagnosis being 66 years. Of note, for African-American men, the occurrence frequency is higher as compared to White men, with 158.3 new cases diagnosed per 100,000 men and their death rate is almost twice as White men^3^. Although there are potential curative options such as radical prostatectomy or radiotherapy, however, once the disease is metastatic, the outlook for the patient is poor. Therefore, it’s crucial to ascertain newer mechanism-based agents and targets to efficiently treat prostate cancer.

Genes particular to the prostate are very significant for the identification and cure of PCa. Proteins belonging (specific) to the prostate can serve as prostate marker proteins for cancer diagnosis, as prostate cancer growth and proliferation is frequently accompanied by aberrant gene expression, which is another cause of prostate cancer. The detection and classification of genes specific to the prostate will be significant to establish the molecular mechanisms for the progression of cancer cells. Furthermore, for therapeutic interventions specificity of prostate cancer can be considered significantly controlled by regulatory processes. According to current research, TPD52 is known as a potential cancer-causing gene (oncogene) located on chromosome 8q21, formerly observed to be amplified and up-regulated in breast cancer. Tumor protein D52 (TPD52) is a highly charged (acidic) mainly present in the peripheral membrane and cytosol which is usually observed to be up-regulated in several cancers^4^. Research on the TPD52 family demonstrates that it may act as novel markers due to its vital role in the proliferation and progression of cancers. It has been verified that in nonmalignant fibroblasts 3T3 (both in vitro and in vivo) the induction of up-regulation of TPD52 and TPD53 causes malignancy and inhibits progression in the cell cycle^5^.

Most cancer-associated mortalities in men are due to prostate cancer. Extensive experimental and financial input has been invested to improve its early detection methods, yet prostate cancer fails to be diagnosed at primary stages. The death rate continues to accelerate in advanced prostate cancer stages, rendering palliative remedies as the sole viable solution. Therefore, to prevent disease occurrence and curb its progression, there is a requirement to come up with an efficient strategy. Plant-derived compounds have garnered wide attention for their clinically advantageous properties against several cancers. These compounds have also been investigated for their anti-tumorigenic influence in prostate cancer. Our team previously demonstrated the antioxidant activity of *Maytenus royleanus* leaves methanol extracts (MEM) and their several fractions that particularly showed recuperative potential against deterioration-related with free-radical generation^6^. The current study reported the pro-apoptotic action of MEM in a model system of prostate carcinoma. Compounds including flavonoids, tannins, and triterpenoids, bound with carbohydrate moieties or polar groups are found to be present in MEM and its fractions. Analysis with known standards further indicated the occurrence of varying quantities of quercetin 3-rhamnoside and caffeic acid in the fractions of MEM. Evaluation of MEM-treated cells through clonogenic survival assays, MTT assay, and time-course analysis revealed the significant attenuation of cell viability, along with G2-phase growth arrest due to up-regulation of CDK inhibitors and decrease expression of cyclins and cdks. Furthermore, enhanced concentrations of cleaved PARP and caspase-3, accompanied by the apoptotic protein network modulation indicated apoptosis induction as a primary anti-carcinogenic mechanism of MEM. In vitro and in vivo prostate cancer model systems showed inhibition of androgen receptor (AR)/ prostate-specific antigen (PSA) signaling. Androgen sensitive CWR22Rν1 cells-xenografted athymic nude mice exhibited a significant reduction in serum concentration of PSA and halted tumor growth, upon intraperitoneal administration of MEM (dosage: 1.25 and 2.5 mg/ animal). In short, the findings of our study demonstrated the therapeutic efficacy of MEM that can be further explored in humans for its potential to curb the progression of prostate cancer^9^. The current study aimed to decode the molecular mechanism of the anti-proliferative effects of MEM by cross-examining the proteomics changes caused by MEM treatment in prostate cancer cells. Label-free nano ESI ultrahigh-resolution mass spectrometry approach was applied for this purpose; employing a Q-Exactive hybrid quadrupole-Orbitrap mass spectrometer. Undeniably, quantitative proteomics united with bioinformatics is an influential way that can be applied to disclose the multifaceted molecular processes in biological setups. In this study, we identified differentially expressed proteins (from a total of 3247), which may be useful in generating a molecular signature of MEM that may be significantly pertinent to prostate cancer.

## Results

### Effect of MEM on the proteome profile of human prostate cancer cell

To determine the effect of MEM on global proteome changes, an effective dose for treatment was determined against two human prostate cell lines C_4-2_ and CWR22Rν1. In our previous published study, MTT assay was performed to investigate the anti-proliferative effect of MEM (0.1, 0.2, 0.5, 1.0, 2.0, 4.0, and 8.0 μM; for 24 and 48 h) in C_4-2_ and CWR22Rν1 cells. After treatment with MEM, both cell lines showed a dose-dependent decrease at OD570 that indicates a reduction in cellular proliferation. A significant decrease in cell growth and cell viability was observed at 1.25 and 2 mg extract concentration. These findings suggest that MEM exerts its cytotoxic effect at 2 μM whereas at lower concentrations (< 1 μM) the drug did not show much effect. Thus, for proteome analysis, 1.25 mg concentration of MEM against CWR22Rν1 cells was selected^7^.

**Figure 1:**
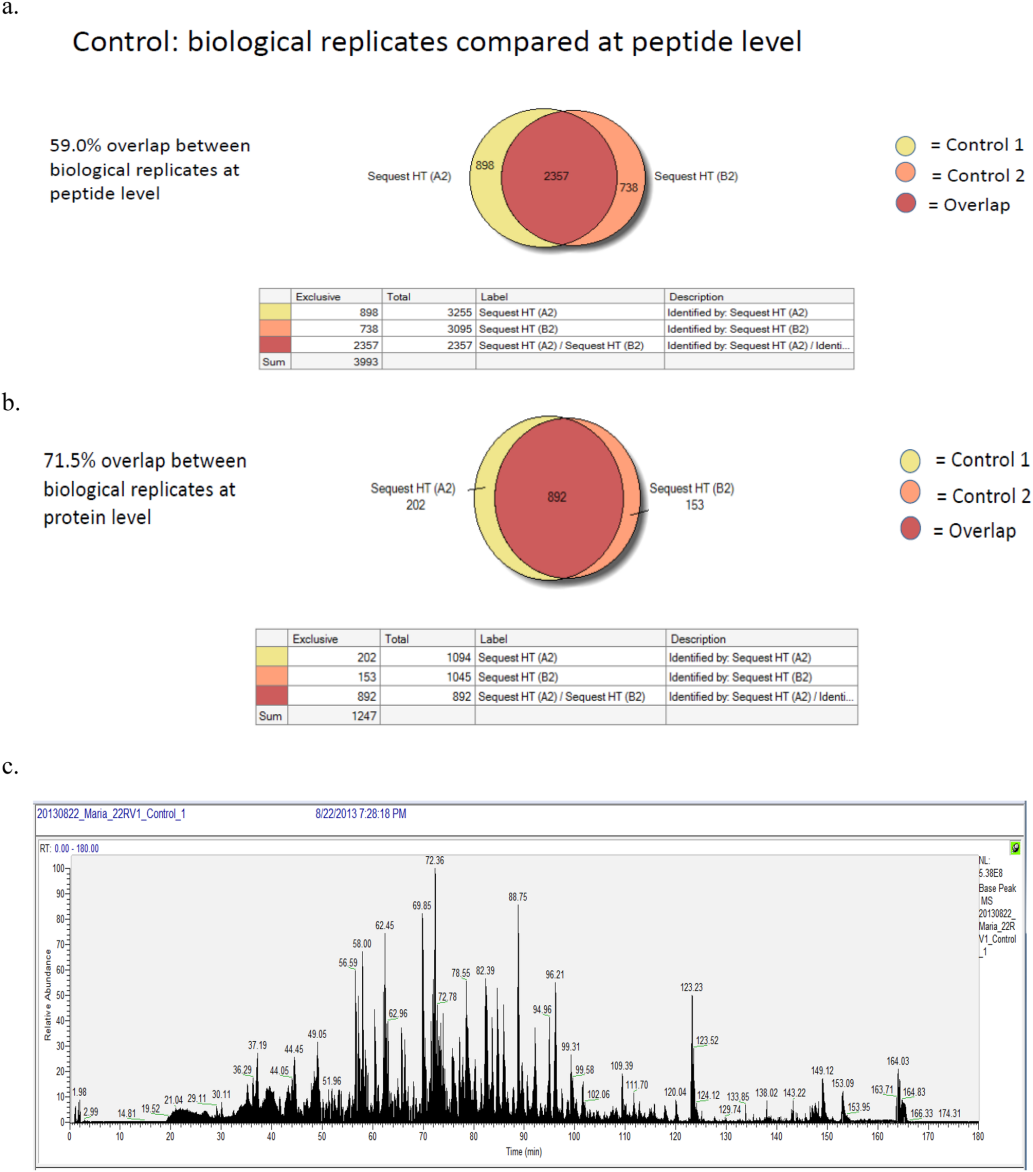

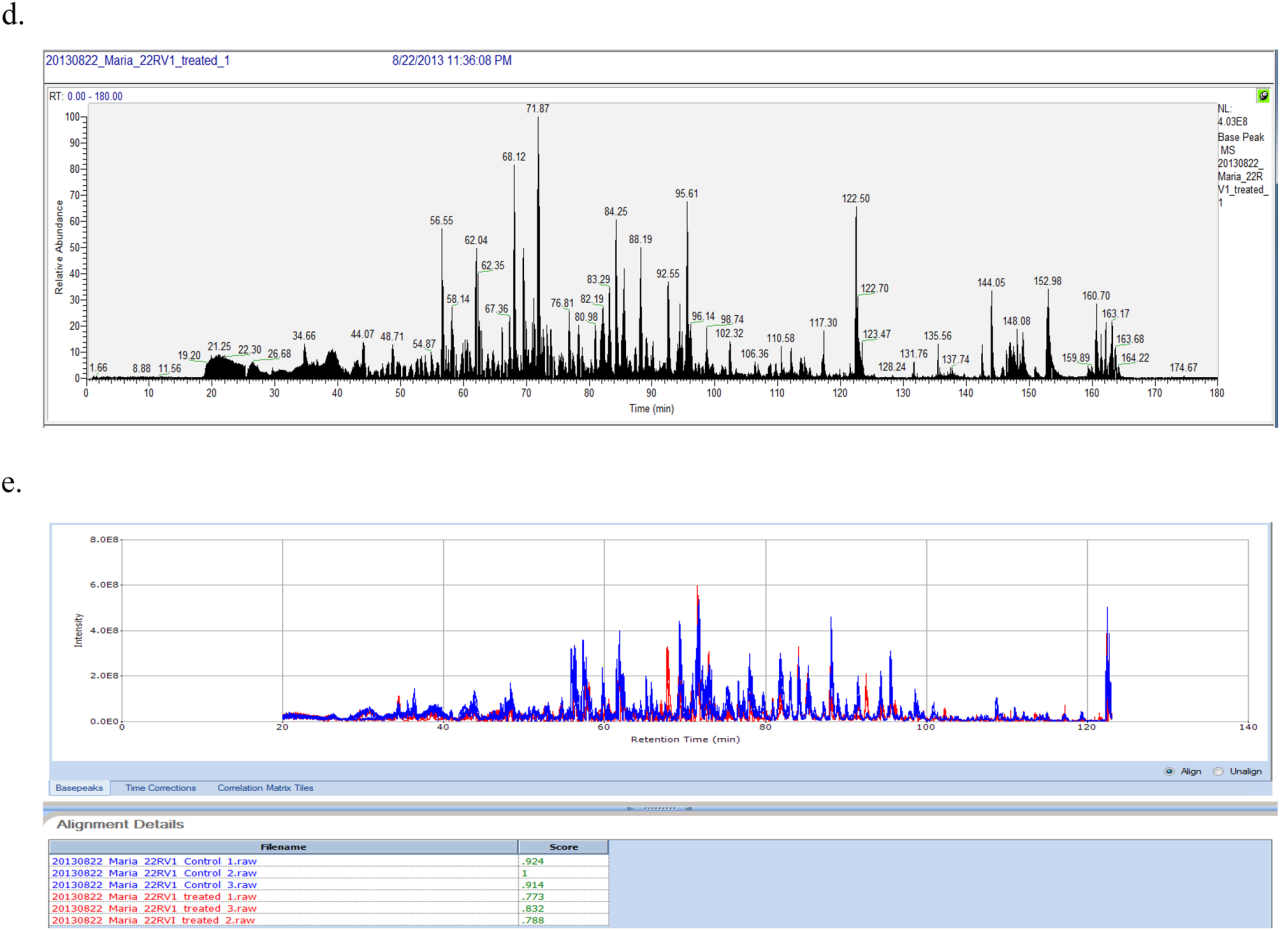
(**a**) Base peak chromatogram of control CWR22Rν1 cells (untreated). (**b**) Base peak chromatogram of MEM treated (40 μg/ml) CWR22Rν1 cells. (**c**) Control biological replicates at the protein level. (**d**) Control biological replicates at the peptide level. (**e**) Overlapping both base peak chromatograms (control + treated) for proteome analysis.

A pre-validation experiment was executed by running duplicate injections of biological replicates that were prepared identically to avoid the data affected by under-sampling. To run sufficient replicates, it is important to have an understanding of the magnitude of under-sampling. When variability between duplicate injections of the same sample with 2% false recovery data (FDR) was compared, a 66% overlap was found. Whereas a 64% overlap was observed between biological replicates at the protein identification level (Fig. 1a,b).

In post-acquisition data processing, the main step is the alignment of the base peak chromatogram. SIEVE 2.1 uses a proprietary algorithm, Chromalign which along with aligning chromatogram region of interest also offers a measure of the quality of the alignment. For valid quantitative analysis, SIEVE needs a score of 0.75 or above whereas our data presented excellent alignment as the scores of control and treated samples were found to be > 0.801. The output data was searched via the Swiss-Prot human proteome database with a decoy using the Sequest HT search engine. It was further evaluated with SIEVE 2.1. for revealing the proteins with altered expression. This analysis identified 3247 proteins at a 0.05 confidence interval. No significant difference was observed in most of the proteins between control and treated samples however less than 5% of the proteins (127 proteins) showed a difference of > 1.5 fold. The data of combined replicates showed an overlap of 59% and 71.5% when compared at peptide and protein levels, respectively. Proteome Discoverer software (Thermo Fisher Scientific Inc.) was used to generate a Venn diagram. The number of proteins in the Venn diagram did not match with the data generated using SIEVE because Proteome Discoverer software re-groups the protein if the peptide is 100% homologous in two proteins, however, SIEVE is not a quantification software and does not know which protein the peptide belongs to and thus expand the protein numbers (Fig. 1c,d,e).

Proteins showing > 1.8-fold change (*p-*value < 0.05) were selected for analysis among the 3247 identified proteins. This criterion was based on the fact that these proteins (> 1.8-fold change) numerically meets the criteria of differentiated proteins however considering fold changes less than 1.8 fail to reach statistical significance during validation. Details such as protein name, protein ID, molecular weight, frames (a defined rectangular region in the m/z vs. retention time plane), number of unique peptides, hits (ms/ms identification scans), % coverage of protein, average p-value and fold change after MEM treatment, are presented in Supplementary data (supplementary file S1).

### Gene ontology analysis of proteome changes

**Figure 2:**
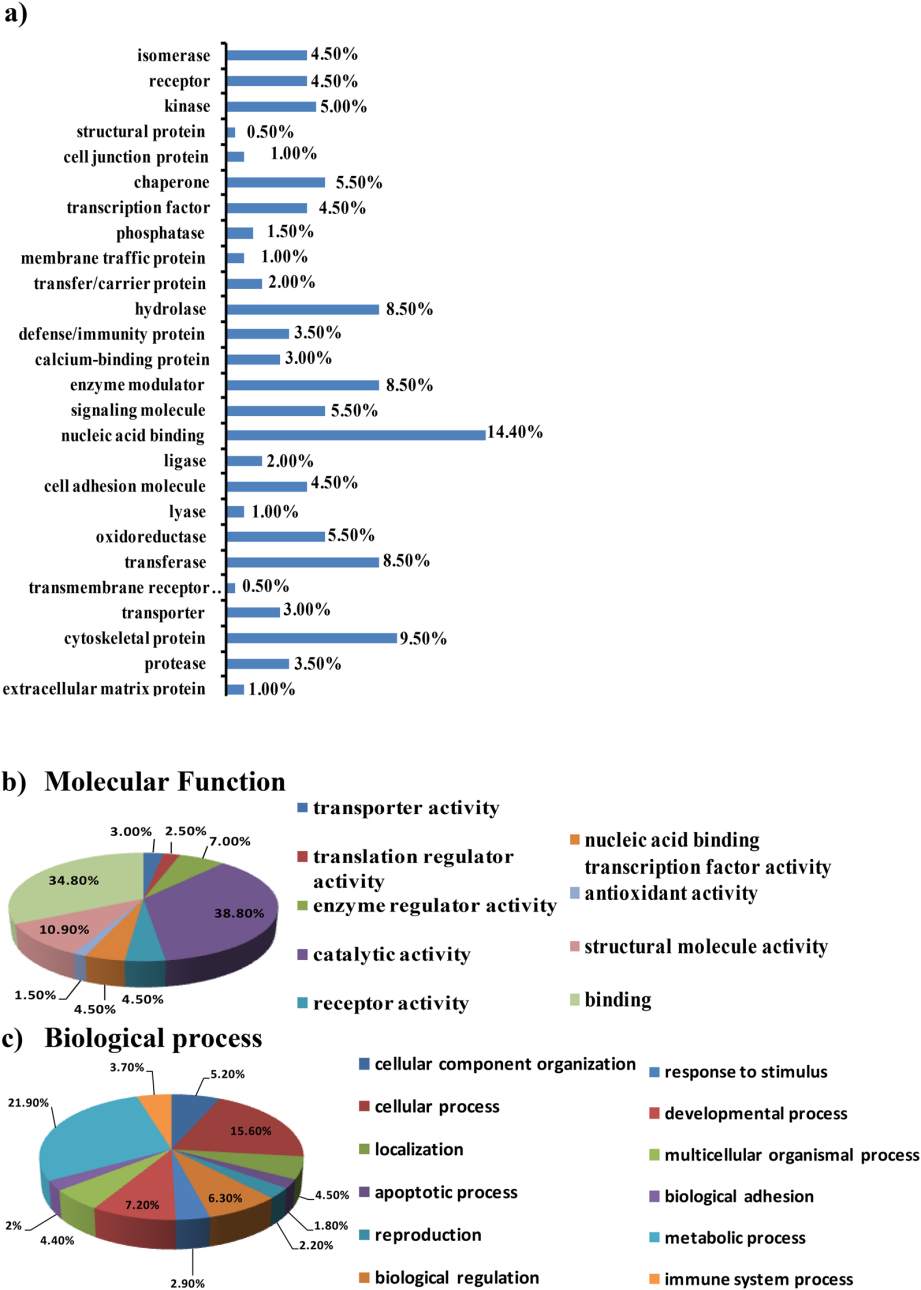
Gene ontology analysis of up and down-regulated proteins. (**a**) Protein functional classification presenting 225 proteins classified in 26 categories. (**b**) Molecular function. 70% MEM treatment-induced proteins. (**c**) Biological process. MEM regulated proteins were found to be involved in 16 biological processes. Gene ontology analysis was performed in the PANTHER database (http;//www.pantherdb.org/).

The selected 30 proteins were elucidated with gene ontology (GO) terms using Protein Analysis Through Evolutionary Relationships (PANTHER) classification system for a better understanding of the biological pathways that were being affected by the transition of protein abundance in response to MEM treatment. The distribution of these proteins among biological processes, molecular functions, and protein classes is exemplified in Fig. 2. Most of the differentially regulated proteins play a significant role in catalytic activity and binding along with minor involvement of transporter activity, nucleic acid binding transcription factor, enzyme regulator, receptor, and functional molecule (Fig. 2a). The major participation of proteins related to metabolic and cellular processes was investigated by GO analysis that shows the molecular events relevant to the proper working of an integrated living system (Fig. 2b). MEM also regulates the immune system, developmental process, cellular component organization, reproduction, apoptotic processes, localization, biological regulation, and multicellular organismal response to the stimulus. MEM affects a vast variety of protein classes such as protease, phosphatase, transferase, cytoskeletal protein, transcription factor, signaling molecules, ligase, enzyme modulators, membrane traffic protein, kinase, and chaperones, therefore, the protein class analysis highlights its pleiotropic mode of action (Fig. 2c). In short, data from gene ontology indicates that MEM strikes numerous critical cellular processes that are related to proliferation and cell growth.

### Pathway analysis by IPA software

Based on fold change and p-value (p = 0.04) TPD52 was selected. After analyzing the 3 biological and 3 technical replicates of both control and treated proteome data, the fold change of TPD52 in treated as compared to control was significant and consistent after each round of analysis in all samples (Supplementary file S1).

**Figure 3:**
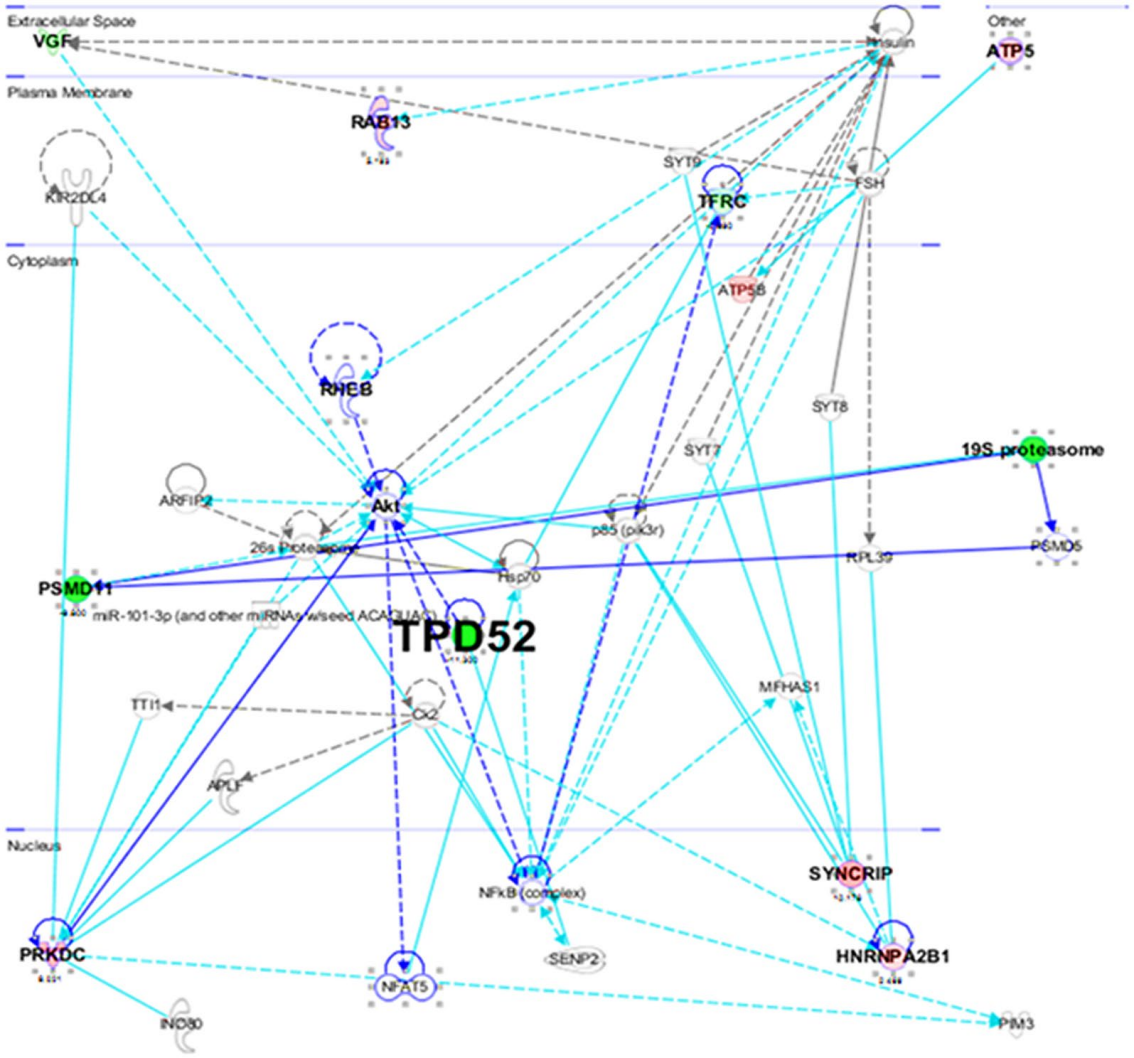
IPA analysis of proteins changing in abundance with MEM treatment. Network analysis by IPA, the molecules involved in the network, and IPA core analysis. The green color shows protein expression that is downregulation and pink represents the upregulated proteins. The network evaluation result from IPA shows yet another sign of the connection between the proteins and cellular processes like apoptosis. IPA demonstrates that many of both up and downregulated proteins in the profile bind and regulate each other and play a common function in oxidative stress. MEM: Methanol extract of *M. royleanus* leaves, IPA: Ingenuity pathway analysis (QIAGEN Inc., https://www.qiagenbioinformatics.com/products/ingenuity-pathway-analysis).

Ingenuity Pathway Analysis (IPA) (QIAGEN Inc., https://www.qiagenbioinformatics.com/products/ingenuitypathway-analysis) executed the putative networks, relationships, and canonical pathway analysis of differentially expressed proteins. To map proteins into biological networks and to recover functions and key pathways the 30 selected proteins were uploaded to the IPA module with their respective Swiss-Prot IDs and corresponding fold changes. The Association of 26 canonical pathways was identified with these selected proteins. Among these, the protein ubiquitination pathway seems to be the most prominent that generally participates in post-translational modifications. The protein ubiquitination system works in a diverse range of cellular processes, including DNA transcription and repair, cell cycle and division, apoptosis, and response to extracellular modulators and stress. The role of various important signaling pathways in the biological response of MEM is identified as shown in Fig. 3.

Next, the involvement of disease and function-related processes were prevised by organizing the MEM-modulated protein network into definite interaction networks. Most of these networks report for biological functions related to cancer, cell-to-cell signaling and interaction, cell morphology, cell cycle, cellular development and cellular maintenance and function, and DNA repair, recombination, and replication as can be seen in supplementary file S1.

Based on their connectivity, the protein–protein networks of MEM-modulated proteins were algorithmically generated. Fisher’s Exact test was used to assess the significant values for pathway and network analyses. Protein–protein networks determined the multiple central nodes, namely JNK, ERK, TPD52, AKT, and MAPK (Supplementary file S1). JNK, ERK, and MAPK are the additional proteins of this network that remained unidentified by the proteomics analysis.

### Validation; TPD52 expression in PCa

**Figure 4:**
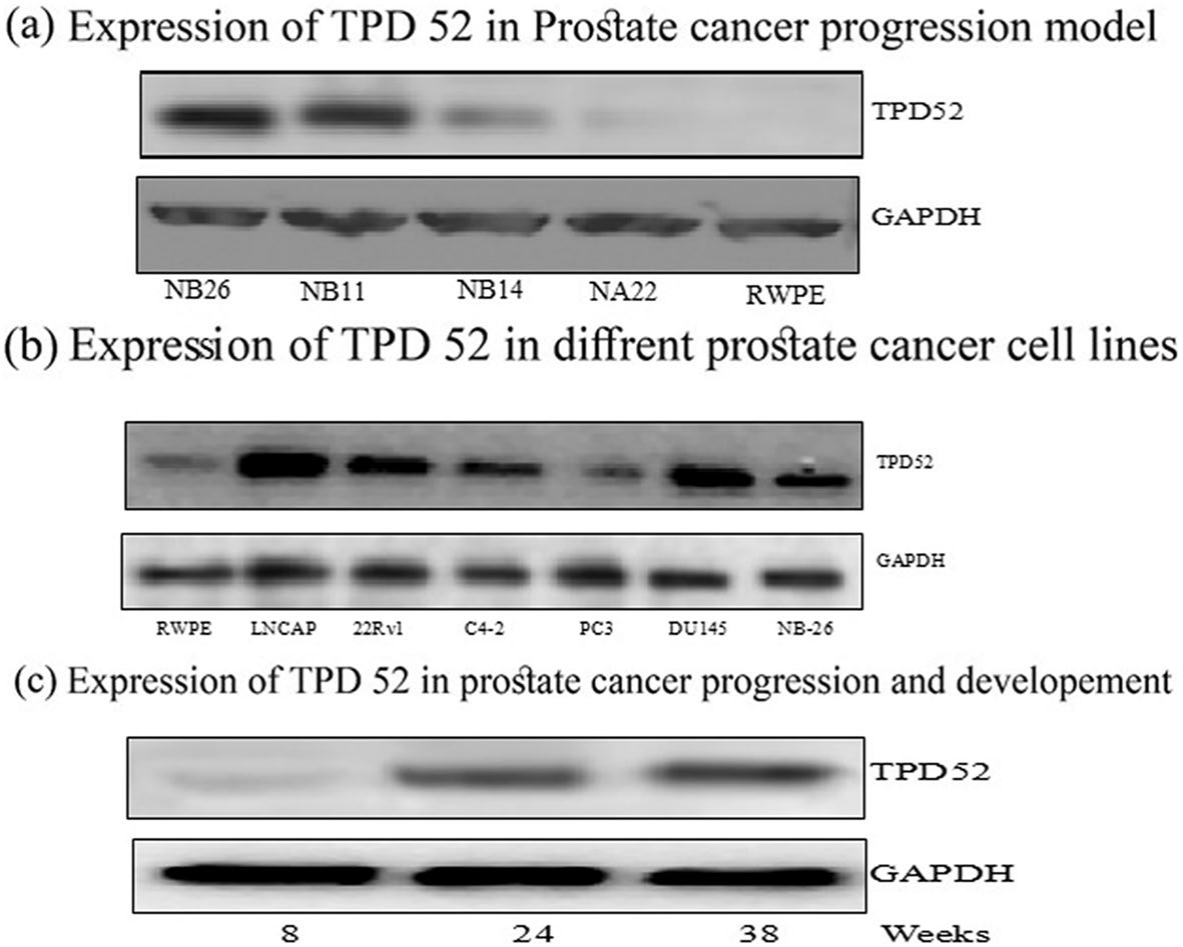
Expression of TPD52 in prostate cancer. (**a**) Expression of TPD52 in prostate cancer progression model. RWPE < WPE2-NA22 < WPE1-NB14 < WPE1-NB11 < WPE1-NB26 with increase in proliferation and progression of cancer protein expression of TPD52 also increased as depicted by Immunoblot analysis. The total cell lysate was prepared and 40 μg protein was subjected to SDS-page followed by Immunoblot analysis and chemiluminescence detection. (**b**) Expression of TPD52 was observed in seven prostate cancer cell lines (RWPE, LnCap, Du145, CWR22Rν1, PC3, C_4-2,_ and NB26) under regular culture conditions. Except for RWPE-1 all other cell lines tested showed marked protein expression of TPD52 in Immunoblot analysis. (**c**) Immunoblot analysis for expression of TPD52 in prostate cancer development and progression model (transgenic adenocarcinoma of the mouse prostate, TRAMP), showed an increase in expression with the development and progression of prostate cancer. The blots shown here are representative of three independent experiments with similar results.

In the current study, we determined the expression of TPD52 in N-methyl nitrosourea (MNU) treated RWPE-1 nontumorigenic cell line which generated a family of tumor cell lines in order of increasing malignancy and invasiveness which is RWPE < WPE2-NA22 < WPE1-NB14 < WPE1-NB11 < WPE1-NB26 (progression model of prostate cancer) described by the laboratory of Webber et al., (2001). It was observed that with an increase in proliferation and progression of cancer the expression of TPD52 protein increases, although there was no expression of TPD52 in RWPE (immortalized cells, nontumorigenic cells) and NB22 cells (least malignant). On the other hand, with an increase in proliferation and progression, the augmentation in the expression of TPD52 was seen in order of NB-14 < NB-11 < NB-26 (Fig. 4a).

Furthermore, expression of TPD52 was observed in all of the tested PCa cell lines (RWPE, LnCap, Du145, CWR22Rν1, PC3, C_4-2,_ and NB26) under regular culture conditions. This was in sharp contrast with both PSA and PSMA well-known prostate cancer markers, which were observed only in the LNCaP (AR expressing) and its lineage derivatives. Whereas TPD52 was homogeneously expressed, its expression was not affected by any type of cell lines neither androgen-independent nor by androgen-dependent cell lines. The evolution from androgen-dependent to androgen-independent status seemed a heterologous process: while LNCaP sublines sustained the ability to control the androgen-responsive gene (TPD52) (Fig. 4b).

Hence, we evaluated the expression of TPD52 in transgenic adenocarcinoma of the mouse prostate (TRAMP) as prostate cancer development and progression have been well characterized in this model. Tramp mice prostate cancer development is endorsed by the expression of the SV40 large and small T antigen and experiences progressive phases of cancer development starting from prostatic intraepithelial neoplasia (PIN) to adenocarcinoma and metastasis. We observed the expression of TPD52 in prostate tissue lysates of TRAMP mice (8, 24, 36 weeks) and the results showed that with the increase of tumor size and growth the expression of TPD52 increased which is again in favor that TPD52 is overexpressed in high stage prostate cancer (Fig. 4c).

**Figure 5:**
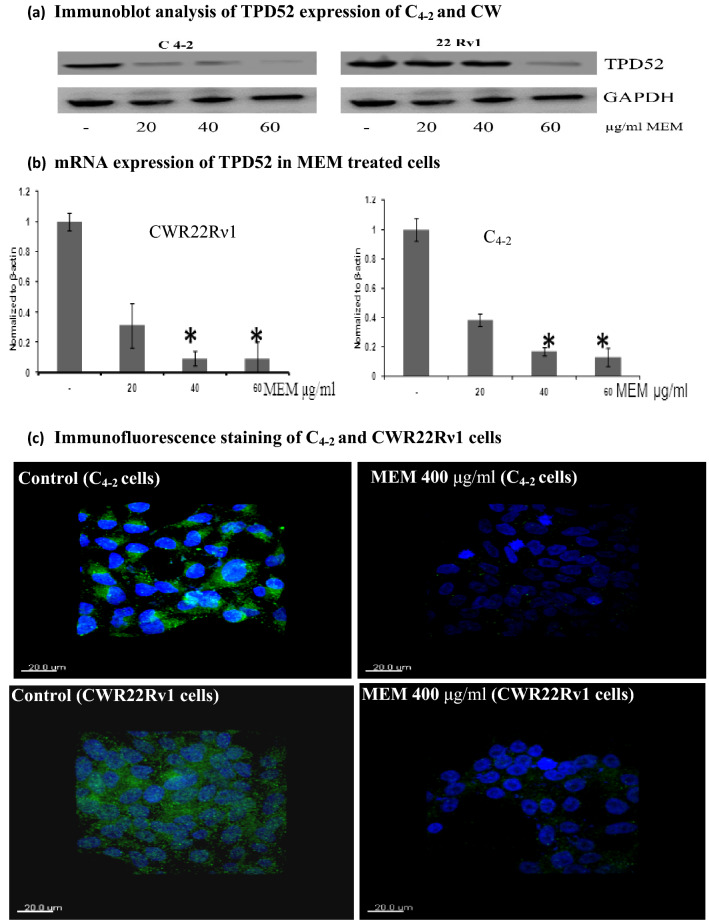
Validation of TPD52 expression in C_4-2_ and CWR22Rν1 cells and effect of MEM treatment on TPD52 expression in both cell lines. (**a**) Immunoblot analysis of TPD52 expression of C_4-2_ and CWR22Rν1 in MEM treated group as compared to the control group. The total cell lysate was prepared and 40 μg protein was subjected to SDS-page followed by Immunoblot analysis and chemiluminescence detection. Equal loading of protein was confirmed by stripping the immunoblot and reprobing it for β-actin, the experiment was done in triplicate. (**b**) Modulation of TPD52 expression by MEM treatment in CWR22Rν1 (b1) and C_4-2_ (b2) cells. mRNA expression of TPD52 in MEM treated CWR22Rν1 and C_4-2_ cells (RT-PCR), experiment performed in triplicate (mean ± SD), *, p < 0.01, **, p < 0.05. (**c**) Immunofluorescence staining of C_4-2_ and CWR22Rν1 cells demonstrating expression of TPD52 in both MEM treated (40 mol/L) of C_4-2_ (c2) and CWR22Rν1 (c4) cells as compared to control C_4-2_ (c1) and CWR22Rν1 (c3) cells (untreated). Alexa fluor staining of TPD52 of both cell lines (green fluorescence) and counterstained with DAPI (blue fluorescence) were observed.

The early research about D52 sequences also shows that these are overexpressed in several human cancers. It was observed that the treatment of CWR22Rν1 and C_4-2_ cells with MEM for 24 h resulted in a significant reduction in the protein expression of TPD52 by immunoblotting, which is in harmony with the status of TPD52 found in *in-vitro* proteome analysis (Fig. 5a).

To test either the observed decrease in TPD52 protein was due to decreased transcription of TPD52 gene, we next evaluated changes of TPD52 expression by MEM treatment in CWR22Rν1 and C_4-2_ cells, a marked reduction in expression of mRNA was found to be in a dose-dependent fashion. At 40 and 60 µg/ml of MEM, a marked reduction in TPD52 expression was seen (Fig. 5b).

Immunofluorescence staining of CWR22Rν1 and C_4-2_ cells showed a decrease in TPD52 expression at a concentration of 40 µg/ml of MEM treated as compared to control (untreated). As our results demonstrate that MEM treatment induced apoptosis and cell cycle arrest by modulation of expression of TPD52 in both PCa cell lines (CWR22Rν1 and C_4-2_ cells) as shown earlier by cleavage of Caspase 3 and PARP, also by modulation of cyclin-dependent kinases in CWR22Rν1 and C_4-2_ cells by treatment of MEM in a dose-dependent manner (Fig. 5c).

**Figure 6:**
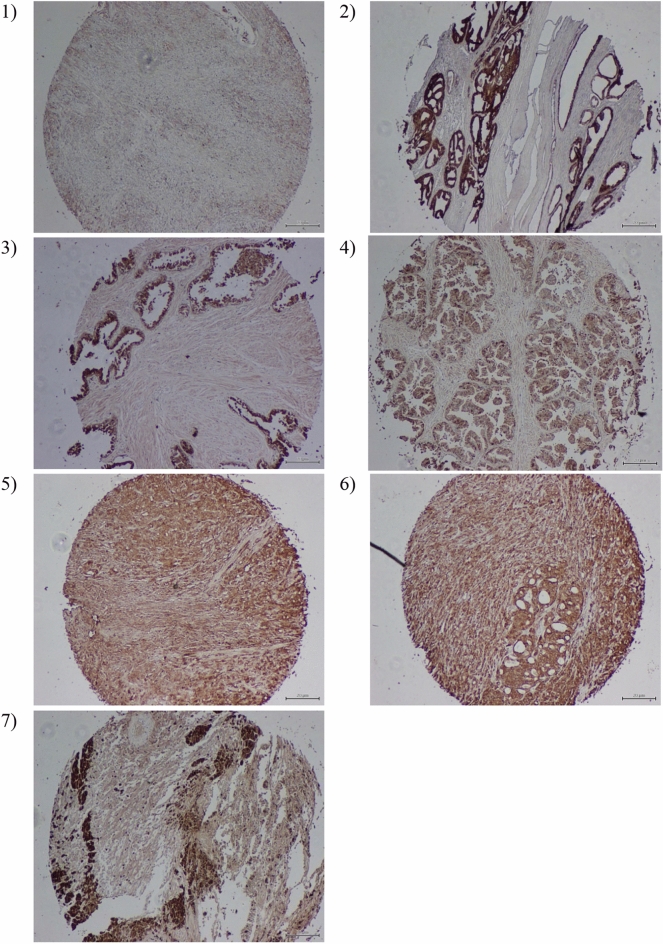
Immunohistochemistry of tissue microarray to validate TPD52 overexpression in PCa. (**A**) Representative data from immunohistochemical studies of 24 prostate cancer specimens are shown. (**A1**) TPD52 expression in a normal, healthy, 33-year-old prostate, with low however noticeable staining observed in glandular epithelia. (**A2**) TPD52 expression in prostate cancer nodules that are heavily stained, benign prostatic hyperplasia in their vicinity was low for TPD52 expression which is comparable with a normal level. (**A3**) TPD52 in high-grade PIN with adjacent normal glands. Epithelia of the high-grade PIN exhibited a general up-regulation of TPD52; adjacent normal or unaffected epithelial cells displayed low-level staining. (**A4**) Uniformly high expression of TPD52 in PCa cells of Gleason scores 3 tumors. (**A5-6**) Representative specimens showed prevalently high TPD52 expression in PCa of type Gleason score 4 tumors. (**A7**) TPD52 staining shown in malignant melanoma section (tissue marker), with high expression of TPD52 in malignant cells of melanoma surrounded by neighboring normal unaffected cells representing low staining of TPD52.

Using the specific antibodies, we determined the expression of TPD52 in association with clinical PCa in a tissue microarray (US Biomax, Inc. T195b serial). Specimens from 24 prostate cancer cases and one case as a tissue marker of malignant melanoma (skin) were used. Tissue microarray contained 21 malignant out of which 2 were low malignant and others represented different stages of malignancy and 4 were normal prostate tissue (Supplementary file S2). TPD52 was in general low in normal secretory epithelia and benign prostatic hyperplasia (BPH) however overexpressed in intraepithelial neoplasia high-grade prostate tissue and prostate cancer. More staining was observed in Gleason grade 4 than Gleason grade 3 tumors. Extremely intense staining of TPD52 was always confined to malignant cells with the adjacent normal epithelial cells with a normal level of staining. High expression of TPD52 was consequently specific to the cell. Therefore, by using a prostate cancer tissue microarray, we were able to differentiate protein expression of TPD52 in a wide variety of prostate samples. Protein expression of TPD52 was cytoplasmic, which was in harmony with previous research in breast cancer^8^. Week to moderate expression of TPD52 was observed in tissue of benign prostate and strong protein expression was seen in clinically localized prostate samples and metastatic prostate cancer. The expression of TPD52 was found to be confined to the cytoplasm and was not seen in the nucleus (Fig. 6).

**Figure 7:**
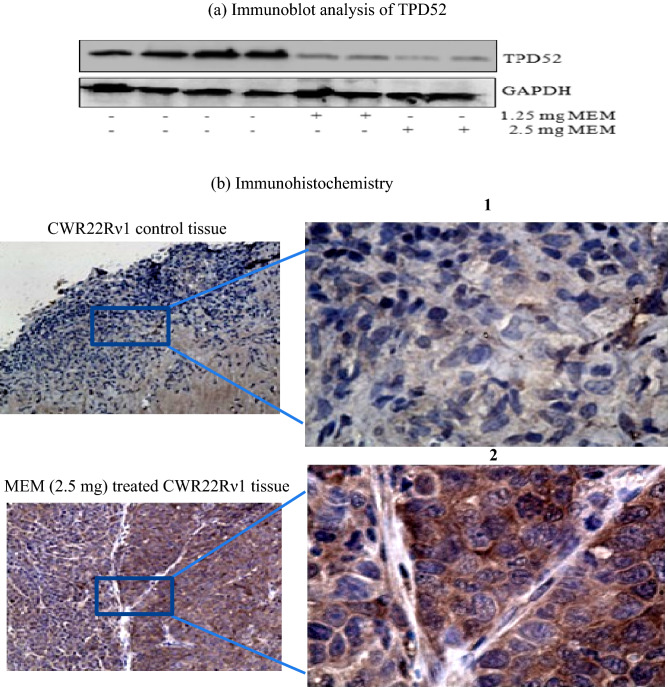
Validation of TPD52 expression in CWR22Rν1 xenograft tissues in athymic nude mice and the effect of MEM treatment on TPD52 expression. (**a**) Immunoblot analysis of TPD52 expression CWR22Rν1 xenograft tissues in MEM treated group as compared to the control group. The total cell lysate was prepared and 40 μg protein was subjected to SDS-page followed by Immunoblot analysis and chemiluminescence detection. The blots shown here are representative of three independent experiments with similar results. (**b**) Immunohistochemistry of CWR22Rν1 xenograft tissues demonstrating expression of TPD52 in control (untreated b.1) and MEM (2.5 mg) treated mice (b.2). An intense brown stain of the cytoplasm of control tissues showed high expression of TPD52 as it is cytoplasmic in origin as compared to MEM treated mice tissue where there is no staining observed.

Depending upon the proteomic outcomes demonstrating that MEM significantly suppresses TPD52 expression, immunoblot analysis (TPD52 protein) of xenografts tumors, control group, and that of treated with MEM was performed for this differentially expressed protein with a specific antibody to validate the levels obtained from data of proteomic analysis. TPD52, protein showed decreased expression significantly after treatment with MEM in xenograft tumors of athymic nude mice as demonstrated by western blot analysis (Fig. 7a).

Results of TPD52 staining showed that there is a decrease in expression of MEM treated CWR22Rν1 athymic nude mice tumor xenografts which resulted in inhibition of proliferation as represented by a decrease in tumor volume by MEM treated groups (Fig. 7c). The control xenografts group (untreated) showed a high expression of TPD52 observed by dark brown staining (DAB) of cytoplasm (Fig. 7b).

### Downregulated TPD52 hampers proliferation, cell invasion, migration, and PSA level in CWR22Rν1 cells

**Figure 8:**
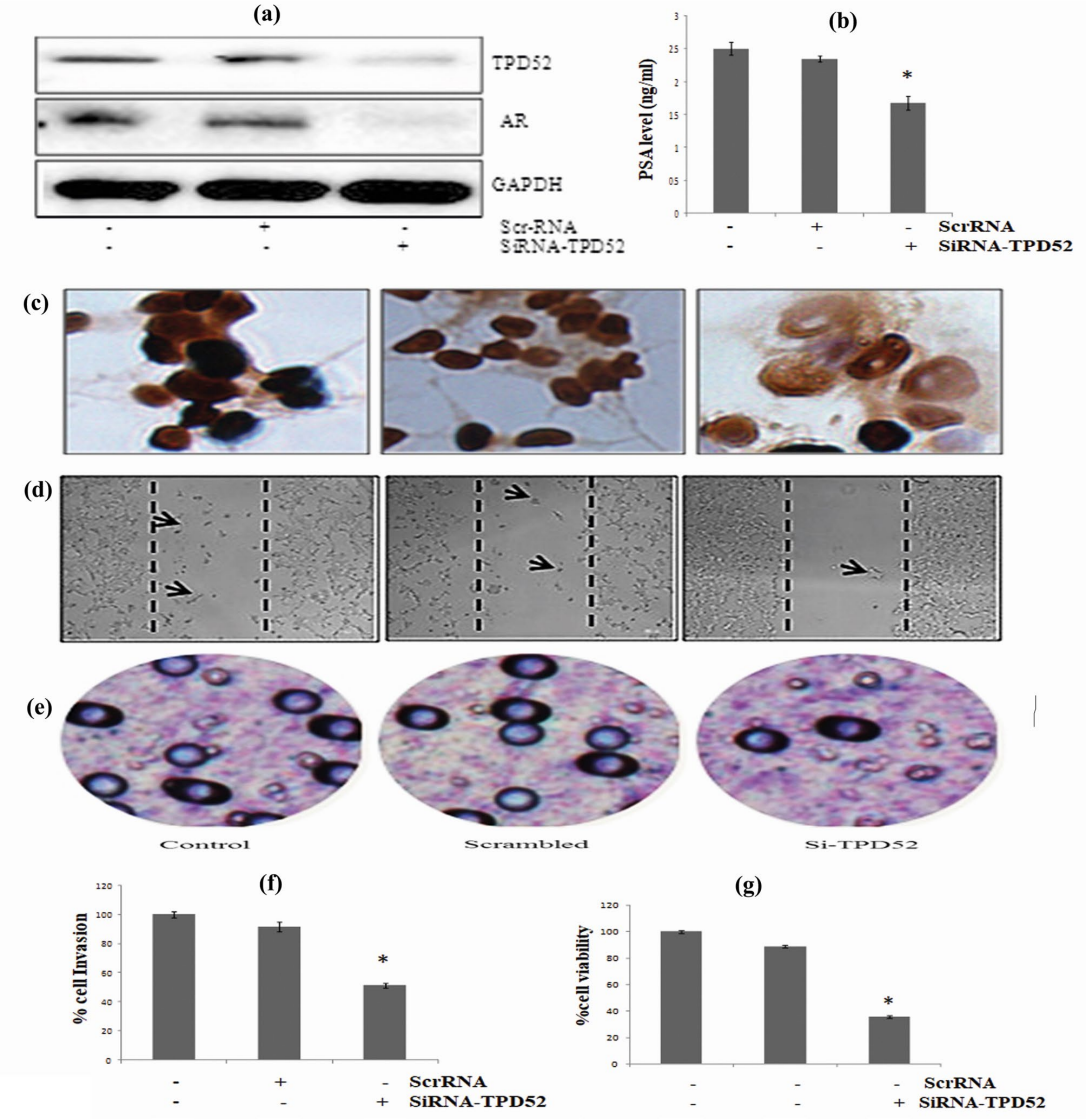
(**a**) Silencing of TPD52 in CWR22Rν1 cells. Western blot analysis of TPD52 protein expression in siRNA-TPD52 transfected CWR22Rν1 cells. The total cell lysate was prepared and 40 μg protein was subjected to SDS-page followed by Immunoblot analysis and chemiluminescence detection. The blots shown here are representative of three independent experiments with similar results. (**b**) Effect of downregulation of TPD52 on the proliferation rate of 22Rv1 cells. 3-(4, 5-dimethylthiazol-2-yl)-2, 5-diphenyl-tetrazolium bromide (MTT) assays were performed after downregulation of TPD52 in 22Rv1 cells. Downregulation of TPD52 leads to decreased cell proliferation as compared to control untransfected and scrambled RNA transfected cells. (**c**) Immunohistochemistry of TPD52-siRNA down-regulated CWR22Rν1 cells. As TPD52 is localized mainly in the cytoplasm so brown (DAB) stain shows an expression of TPD52 and the blue counterstain (hematoxylin) represents the nucleus. The first left represents control untransfected CWR22Rν1 cells showing high staining of TPD52, the middle is scrambled RNA transfected cells also show staining close to normal cells and the third is SiRNA-TPD52 transfected CWR22Rν1 cells showing low to mild staining of TPD52. (**d**) Migration of TPD52-siRNA down-regulated CWR22Rν1 cells. As shown by microscopic analysis, the first left figure represents untransfected normal CWR22Rν1 cells showing almost 60% of migrated cells, the middle figure represents scrambled RNA transfected cells showing almost the same number of migrated cells as that of normal control and third is SiRNA-TPD52 transfected CWR22Rν1 cells which show only 10% of migrating cells.

We validated whether MEM induced downregulation of TPD52 causes induction of apoptosis and decrease in proliferation (downregulation) was regulated by TPD52 signaling, Transfection of 22Rv1 cells with Si-RNA of TPD52 designed to downregulate TPD52 expression in 22Rv1 cells was carried out. A considerable downregulation was seen at the protein level, as validated by western blot analysis, corresponding bands showed a decrease in expression of TPD52 as compared to that of the control level (Fig. 8a). No marked variation was seen between untransfected and scrambled RNA transfected cells. Our data demonstrated that these effects are mediated in part by TPD52 and other means of actions are also involved (Fig. 8b). Our observation of the effect of down-regulated TPD52 in the proliferation of CWR22Rν1 cells came from an MMT assay. Downregulation of TPD52 leads to reduced cell proliferation as compared to control untransfected and scrambled RNA transfected cells.

To determine the effect of downregulation of TPD52 on the invasiveness of PCa cells, standard *invitro* chamber assays with a matrigel model were performed. As shown in the figure the number of cells that digested and penetrated Matrigel through the trans-well polycarbonate filter was highly reduced by siRNA-TPD52 transfection. Compared with the Si-RNA scrambled transfected cells and parental cells, SiRNA-TPD52 transfected cells reduced invasiveness at 24 h post-transfection (p = 0.021) (Fig. 8b).

Immunohistochemical staining of transfected CWR22Rν1 cells showed a decrease in TPD52 expression as compared to control (untreated) and scrambled RNA transfected cells (Fig. 8c).

Moreover, we observed whether the downregulation of TPD52 affects CWR22Rν1 cell migration. We used a scratch wound healing assay of tissue culture monolayers to a 6 well plate format. By scratching the cell-substrate mechanically with a pin, we are capable to generate natural sized wounds in all wells of a 6 well plate. Imaging of the healing wounds with a microscope allows us to differentiate perturbations that affect cell migration. There were minimal to no cell migration observed in downregulated CWR22Rν1 cells as compared to untreated control (untransfected) and scrambled RNA transfected CWR22Rν1 cells which showed marked migration after 24-h incubation. The migration potential of cancer cells is essential for the development of metastasis. Downregulation of TPD52 inhibited the migration of prostate cancer cells (Fig. 8d).

**Figure 9:**
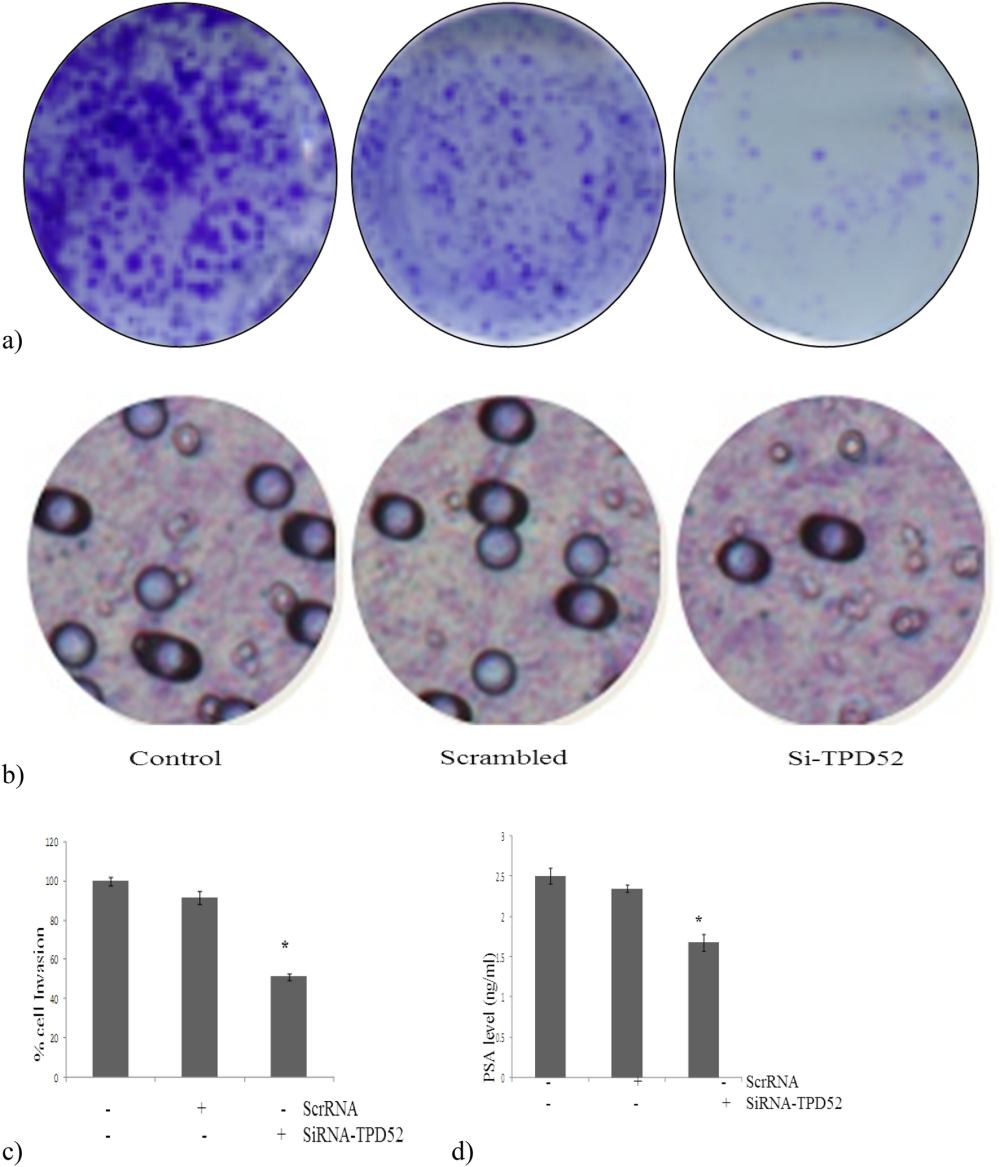
(**a**) TPD52-siRNA down-regulated colony formation ability of CWR22Rν1 cells in colony formation assay. (**b**) TPD52-siRNA down-regulated invasion property of CWR22Rν1 cells. Microscopic analysis showed that the first left figure represents untransfected normal CWR22Rν1 cells showing almost 55% of invaded cells, the middle figure represents scrambled RNA transfected cells showing almost the same number of invasive cells as that of normal control and third is SiRNA-TPD52 transfected CWR22Rν1 cells which show only 10% of cell invasion. (**c**) Quantitative analysis of cell invasion by dissolving stained cells in 10% acetic acid (100–200 μl/well) and transfer a consistent amount of the dye/solute mixture to a 96 well plate for a calorimetric reading of OD at 560 nm (p < 0.021). (**d**) ELIZA for serum PSA secretion of TPD52-siRNA down-regulated CWR22Rν1 cells. Effect of TPD52-siRNA down-regulated CWR22Rν1 cells on serum PSA secretion in media (p < 0.0032). All experiments (A-F) were done in triplicate.

Furthermore, we observed that the siRNA-TPD52 transfection of CWR22Rν1 cells resulted in inhibition of tumor growth, cell migration as well as a reduction in the colony-forming ability with a marked reduction in the secretion of prostate-specific antigen (PSA) in the serum. To determine PSA levels in siRNA-TPD52 transfected CWR22Rν1 cells, a quantitative sandwich ELISA technique was used. Downregulation of TPD52, significant inhibition of PSA expression was observed as compared to normal control cells (nontransfected) and that scrambled RNA transfected CWR22Rν1 cells. Thus our results clearly showed that downregulation of TPD52 demonstrated a marked reduction (p < 0.0032) in PSA secretion of cells. Hence, the effects of the downregulation of TPD52 on the PSA level of cells correlate with inhibition of growth and proliferation of prostate cancer cells (Fig. 9a–d).

## Discussion

To observe the molecular alterations that generate these phenotypic and malignant variations, proteomic analysis is now being applied to estimate variations in protein expression, alterations, and enzyme activity. Furthermore, identifying significant proteins and their modified regulatory functions provides in-depth knowledge about the evolutionary process of tumor cells which reveals new functions and phenotypes. The uncertainty in using a specific single protein as a biomarker for a disorder has paved the way for the development of a series of proteins (biomarkers) instead of a single protein for various diseases. An additional significance of proteomics is to determine the molecular methodologies and signaling cascades that cause the development of cancer. On the whole proteomics analysis can support the development of cancer research for the Biomarker discovery of cancer for the development of molecular detection for therapeutic interventions, Proteomics offers an improved perceptive of the molecular pathology of cancer (cell signaling), Adapted cancer therapy, (Drug targeting, assisting the integration of diagnostic and therapeutic characteristic of cancer), improved categorization of cancer, Toxiproteomics, that might assist in the development of safer treatments for cancer by exploring toxic side effects of anticancer drugs at a premature stage and patient monitoring.

After iTRAQ analyses (three biological replicates), we consistently identified 1000 proteins. All the proteins identified were most abundant in the CWR22Rν1 cell proteome. Among these proteins, many proteins had significantly altered expression because of MEM treatment. The proteins identified in proteome analysis are mostly involved in the synthesis and transcription of DNA, redox balance, cell death, the formation and alteration of ionic transport in the cytoskeleton. The enhanced discharge of cytochrome c and various apoptosis promoting molecules might suggest that cellular death may be stimulated by activation of the Caspase network and induced by Reactive oxygen species. Pathway determination by IPA software offers an additional sign for the association among proteins involved in these networks and processes like cell death. Ingenuity pathway analysis (IPA) verifies that most of the proteins (up or down-regulated) control the expression of each other and take part in reactive oxygen species-induced stress. Amongst the known proteins we chose those proteins that had an altered expression caused by the treatment of MEM, mainly those responsible for prostate cancer proliferation. According to IPA analysis, the five top canonical pathways which were affected by the treatment of methanol extract of *M. royleanus* leaves were (1), 14–3-3 mediated signaling (p valve, 1.97 × 10^–05^). (2), Protein ubiquitination pathway (P valve, 4.35 × 10^–05^). (3), p70S6K Signaling (p-value, 1.79 × 10^–04^). (4), PI3K/AKT signaling (p-value, 2.08 × 10^–04^). (5), Protein kinase A signaling (3.72 × 10^–04^).

The 14-3-3 proteins are a dimeric, highly conserved family of protein present in all eukaryotic organisms^9^. In humans, seven isoforms of 14-3-3 proteins have been recognized (Aitken, 2006). 14-3-3 proteins (29–31 kDa) form a complex with target proteins on phosphorylated serine/threonine subunits^10, 11^. Consequently, 14-3-3 proteins actively control numerous signaling such as tumor growth and pathways that regulate various biological processes. It is also well known that 14-3-3 proteins hinder cell death^12^. They act as a positive control of the Akt downstream pathway in reaction to survival signals by forming complex and quenching the apoptosis promoting molecules (Bad) apart from their interacting associates and places where they act^12, 13^. They have also been known to cooperate with other endurance endorsing molecules like PI3K (phosphoinositide 3-kinase) and growth factor receptors^14, 15^. On the other hand, the outcomes of these contacts particularly associated with the proliferation of cancer cells are still unclear^13, 16^. In response to stimuli mainly through receptor tyrosine kinases (PI3Ks) control various cellular processes the same as 14-3-3 proteins including cell death^17^. Activation of PI3K encourages phospholipid substrates accumulation in the membrane and causes activation of downstream signaling cascades including AKT signaling that regulates cell growth and survival. In various cancers upregulation of the PI3K/ or protein kinase B signaling cascade has been known and is linked with enhanced progression and proliferation of cancer cell. Improved knowledge of the regulatory processes that cause activation of PI3K will convey vital information about Phosphoinositide 3-kinase signaling in both cancerous and normal cells that might assist in the progress of effective targeting. A multicatalytic enzyme complex (2.5MDa) such as proteasome contains a 20S catalytic core and two 19S regulatory subunits. Since, proteasome targets a lot of proteins that are involved in the control of important cascades of cancer and cancer cell survival (cell cycle progression, proliferation, differentiation, and cell death) its inhibition can cause cell death or growth arrest. Consequently, in the past research has been dedicated to screening inhibitors of proteasome for cancer therapy.

Protein expression (Ingenuity pathway analysis) in normal control and MEM treated determined that the protein with the top scores is responsible for amino acid metabolism, behavior, and protein degradation. The nuclear factor kappa- light chain enhancer of activated B cells (NF-кB) and extracellular signaling; protein kinase cascades (mitogen-activated) in this pathway were related to protein kinase B, protein kinase C isoforms, insulin, and PI3k expressions were not altered. It means that MEM treatment extract can also reduce the expression of NF-кB in both in vivo and *invitro* systems. Overall, proteome analysis by MALDI–TOF–MS and ingenuity pathway analysis revealed main proteins and signaling pathways that may be liable for inhibitory effects of MEM treatment of CWR22Rν1 in both in vivo and in vitro systems. The protein expression in cells treated with MEM in both in vivo xenografts and in vitro models was compared with each other and also with controls of both (untreated).

Amongst the proteins with downregulated expression after MEM treatment, the expression of TPD52, PCNA, and PSMA was noteworthy. Proteome analysis results are in harmony with the study that determined the overexpression of TPD52 protein in PCa cancer biopsies through proteome analysis. So far the major role of tumor protein D52 (TPD52) in PCa proliferation is still not clear^18^. Recent research demonstrated that TPD52 was up-regulated, translocates from early to adult tissues during development, its expression mainly depends on age^19^.

A series of molecular processes playing a vital role in PCa proliferation, genetic changes like a mutation in chromosome 8 at 8q21 consist of important abnormalities causing prostate cancer. The altered expression of a gene that was further allocated as tumor protein D52 having locus on chromosome 8q21 was indicated by cDNA library analysis. The gene encoded by TPD52 is also known as PrLZ is recognized as a proto-oncogene and is a part of the tumor protein D52 (TPD52 gene/protein family) family. Tumor protein D52 is up-regulated in prostate cancer, breast cancer, and also in ovarian cancer due to gene amplification. Tumor protein D52 gene classification study observed that TPD52 is expressed dominantly in epithelial cells and plays a significant task in their phenotype. In LNCaP cells, the expression of TPD52 protein is regulated mainly by androgens. The androgen receptor is activated by circulating androgen (testosterone) to regulate various processes of cells like uncontrolled growth, cell death, and other metabolic processes in PCa. As a whole, the interaction of genes, amplification, and stimulation of androgen may induce the upregulation of tumor protein D52 in PCa. Tumor protein D52 goes through alterations (post-translational, e.g. phosphorylation), in a calcium reliant fashion that interacts with annexin VI and MAL2. TPD52 (Murine) causes the progression of NIH3T3 fibroblasts and tumor formation. Current research shows that the PrLZ gene expression is upregulated and activated during cancer development and proliferation from early to later stages of tumor that causes activation of the protein kinase B cascade that performs a vital task in the progression of PCa. Since the exact role of Tumor protein D52 in proliferation and progression of PCa is not clear yet, the aim of the current study was the functional categorization of Tumor protein D52 changes in PCa cell line CWR22Rν1 (androgen-responsive). The role of tumor protein D52 was examined in various cellular events in PCa^20^.

TPD52 is a recently known prostate-specific and androgen inducible gene. Since it is upregulated in CWR22Rν1 cells and overexpressed in human PCa, we hypothesized that TPD52 may take part in the growth and proliferation of androgen-independent prostate cancer. Similar to in vitro data, intraperitoneal injection of MEM considerably slowed tumor growth in athymic mice, inhibited TPD52 expression, and resulted in a considerable reduction in the level of PSA in the serum. Depending upon the proteomic outcomes demonstrating that MEM significantly suppresses TPD52 expression, we demonstrated whether MEM alters rates of TPD52 protein degradation. Immunoblot analysis (TPD52 protein) of xenografts tumors (same for which proteomic analysis was done) control group and that of treated with MEM was performed for this differentially expressed protein with a specific antibody to validate the levels obtained from data of proteomic analysis. GAPDH was used as a reference for the comparison of protein levels. TPD52, protein has a high level of expression in prostate cancer and the expression decreased significantly after treatment with MEM in xenograft tumors of athymic nude mice as demonstrated by western blot analysis. To further investigate the potential oncogenic properties of TPD52 in CWR22Rν1 xenograft tumors, immunohistochemical analysis was done to investigate TPD52 in paraffin-embedded tissues. Results of TPD52 staining showed that there is a decrease in expression of these proteins in MEM treated CWR22Rν1 athymic nude mice tumor xenografts resulted in inhibition of proliferation as represented by a decrease in tumor volume by MEM treated groups. Control xenografts group (untreated) showed high expression of TPD52 observed by dark brown staining (DAB) of cytoplasm; our results are in harmony with the findings of Ummanni et al. (2008) according to him a proteomic analysis of prostate biopsies presented overexpression of TPD52 at the proteome level^21^.

Research about tumor protein D52 (sequences) also elucidates that they are up-regulated in numerous human carcinomas. To further validate that MEM harms the expression of TPD52, we conducted a western blot analysis. A marked reduction in the expression of TPD52 protein was observed, which is in harmony with the status of TPD52 found in vitro proteome analysis. To test either the observed decrease in TPD52 protein was due to decreased transcription of the TPD52 gene, we next examined alteration in TPD52 protein expression by MEM induction in CWR22Rν1 and C_4-2_ cells, a marked reduction in expression of mRNA was observed with .an increase in dose. Evidence shows that TPD52 is a well-known androgen-responsive gene that is significantly expressed in prostatic tissue. The CWR22Rν1 and C_4-2_ cells show upregulated expression of intracellular TPD52 protein, as confirmed by a 24-kDa band. Immunofluorescence staining of CWR22Rν1 and C_4-2_ cells showed a decrease in TPD52 expression at a concentration of 40 µg/ml of MEM treated as compared to control (untreated). As our results demonstrate that MEM treatment induced apoptosis and cell cycle arrest by modulation of TPD52 expression in both PCa cell lines (CWR22Rν1 and C_4-2_ cells) shown earlier by cleavage of Caspase 3 and PARP, also by modulation of cyclin-dependent kinases in CWR22Rν1 and C_4-2_ cells by treatment of MEM in a dose-dependent fashion.

To generate the RWPE-1 cell line (non-tumorigenic), cells (Epithelial) extracted from the peripheral region of a normal adult prostate (histologically) were altered by a distinct copy of the HPV-18 (human papillomavirus 18) (Bello et al., 1997). N-methyl N nitrosourea (MNU) exposure to RWPE-1 cells produced a series of progressive tumor cell lines that showed an increase in invasion potential (NA22, NB11, and NB26). These series of cell lines with the same lineage show a distinctive and related system that copies different phases of proliferation from the tumor (localized) to cancer that is invasive and usually used to study cancer, proliferation, prevention, and treatment^22^. The present research demonstrated the expression of TPD52 in tumor cell lines in order of increasing malignancy and invasiveness which is RWPE < NA22 < NB14 < NB11 < NB26 (progression model of prostate cancer) described by the laboratory of Webber (2001). It was observed that with the increase in proliferation and progression of cancer the expression of TPD52 protein increases, although there was no expression of TPD52 in RWPE (immortalized cells, nontumorigenic cells) and NB22 cells (least malignant). However, with an increase in proliferation and progression the boost in TPD52 expression was determined in order of NB-14 < NB11 < NB-26. A study on TPD52 gene categorization demonstrated that TPD52 is expressed by epithelial cells and it might contribute to the phenotypic growth of epithelial cells (Chen et al., 1997).

While TPD52 was expressed consistently its expression was not affected by any kind of cell lines whether it is androgen-independent or by androgen-dependent cell lines. The shift from androgen-dependent to androgen-independent condition seemed to be a heterologous process: while LNCaP sublines uphold the potential of controlling androgen-responsive genes (TPD52). Moreover, Expression of TPD52 was observed in all of the tested prostate cancer cell lines (RWPE, C_4-2_, Du145, CWR22Rν1, PC3, LnCap, and NB26) under regular culture conditions. This was in comparison with both PSA and PSMA well-known prostate cancer markers, which were observed only in the LNCaP (AR expressing) and its lineage derivatives.

The TRAMP model (transgenic adenocarcinoma of mouse prostate) is an aggressive model of prostate cancer^23^. Normally TRAMP mice generate low-grade and non-invasive prostate cancer by the 10th week of age^24^. The tumor progresses as mice develop low grade non-invasive to high-grade invasive adenocarcinoma and consequently weakly differentiated neuroendocrine tumors (20–25 weeks of age). The progressive character of the TRAMP model demonstrates a chance to evaluate the potential of chemopreventive agents for both the initial and late stages of prostate cancer development. Prostate cancer death occurs in men with high-grade late-stage cancer; we tried to observe the oncogenic expression of TPD52 in the TRAMP model. These studies observed the upregulation of TPD52 expression with the development and progression of prostate cancer.

Our analyses of the tissue microarray collection of 25 specimens confirmed the clinical significance of our findings identifying TPD52 as a potential marker for PCa progression. Immunohistochemical analysis of a large group of prostate cancers showed that marked TPD52 overexpression occurred in virtually all grades from early-stage prostate cancer to high-grade prostate cancer. These findings reveal that TPD52 overexpression is a potential molecular marker with high sensitivity for prostate cancer. TPD52 expression is related to cell growth progression in various types of cancer cells. Research showed that the TPD52 expression by transduction of retrovirus in neuroepithelial cells showed its part in cellular progression^4, 25^.

High expression of anti-apoptotic proteins or the downregulation of apoptosis promoting proteins is a well-known molecular approach used by tumor cells to prevent cell death. Human D53L1 members of the TPD52 family act together with an apoptotic signal which regulates Kinase I and promotes cell death^26^. RNA interference technique used to silence genes consists of useful tools to verify proteins under investigation^27, 28^. The present study demonstrated that TPD52 knockdown CWR22Rν1 cells are accompanied by an increased rate of apoptosis, verified by MTT cell viability assay, our results are in harmony with Ummanni et al*.* (2008) who demonstrated that knockdown of TPD52 in LnCap cells is escorted by an increased rate of apoptosis. A considerable downregulation was seen at the protein level, as validated by immunoblot analysis, corresponding bands demonstrated a reduction in TPD52 expression as compared to that of the control level. No marked difference was seen among untransfected and cells transfected with scrambled RNA. Our data demonstrated that these effects are mediated in part by TPD52 and other means of action are also involved.

It has been anticipated that the formation of cancer is because of numerous molecular processes that lead to the conversion of healthy cells into tumor cells leading to metastasis and progression, as well as cell invasion into the adjacent tissue, endurance, and progression in the host tissue^29^. The murine TPD52 expression in NIH3T3 cells enhances the expression of several genes concerned with the progression of the tumor to the last stage and the genes involved in impediment of carcinogenesis were downregulated^5^. Tumor cells are oftentimes distinguished as malignant because of their capacity to invade and migrate to distant sites through traversing basement membranes and connective tissues. This process initiates with the abrogation of cell-to-cell and cell-to-ECM communication. Mechanistically, the aberrant concentration of uPA is reported to promote signaling through the cell’s pro-survival pathways such as Akt and MAPK31. Based on this literature, we hypothesized the regulatory influence of MEM on AKT signaling cascade that is demonstrated to be crucial for modulating tumor cell invasiveness^9^. Our cell migration assays observed whether the downregulation of TPD52/AKT affects CWR22Rν1 cell migration. Scratch wound healing assay was performed in a 6 well plate format of tissue culture (monolayers). By scraping the cells mechanically using a pin, wounds are generated in all wells of a 6 well plate. Image of the healing wounds with the help of a microscope permits us to differentiate disturbance that affects the migration of the cell. There were minimal to no cell migration observed in downregulated CWR22Rν1 cells as compared to untreated control (untransfected) and scrambled RNA transfected CWR22Rν1 cells which showed marked migration after 24 h incubation. The migration potential of cancer cells is essential for the development of metastasis. Downregulation of TPD52 inhibited the migration of prostate cancer cells. The same results were observed when we determined the consequence of downregulation of TPD52 on the invasiveness of prostate cancer cells, (standard in vitro chamber assays with a matrigel model were performed). Compared with the Si-RNA scrambled transfected cells and parental cells, SiRNA-TPD52 transfected cells reduced invasiveness at 24 h post-transfection.

Research shows that both estradiol and androgen affect the tumor protein D52 expression which is considered as vital signaling molecules in breast and prostate cancer respectively. Evaluation of protein expression of prostate biopsies discovered variably expressed proteins in cancer comprising of various proteins that are acknowledged as deregulated in PCa ^21^. Moreover, we observed that the siRNA-TPD52 transfection of CWR22Rν1 cells resulted in tumor growth inhibition with a considerable reduction in the secretion of PSA (prostate-specific antigen) in the serum. To determine PSA levels in siRNA-TPD52 transfected CWR22Rν1 cells, a quantitative sandwich ELISA technique was used. Downregulation of TPD52, significant inhibition of PSA expression was observed as compared to normal control cells (nontransfected) and that scrambled RNA transfected CWR22Rν1 cells.

Research has demonstrated that TPD52 expression may be controlled partly by androgens, as demonstrated by a study from De Primo et al. (2002) and Nelson et al. (2002)^30, 31^. Besides, TPD52 might up-regulate the PSA an acknowledged downstream target gene of the androgen receptor which showed TPD52 mediated the tumor growth of prostate cancer was related to signals of androgen.

## Conclusions

Overall, proteomic profiling revealed the main signaling pathways and proteins that are perhaps responsible for inhibitory effects induced by the treatment of MEM extract on CWR22Rν1 cells (in vivo and in vitro systems). This report illustrates a molecular overview of pathological processes in PCa, indicating possible new disease biomarkers and therapeutic targets.

## Methods

### Collection of plant

Leaves of *M. royleanus* were collected from village Lehtrar, Tehsil Kotli Sattian, District Rawalpindi, Pakistan in March 2014. The local name was used for the identification of the plant and then verified by Doctor Saleem Ahmad (curator at Herbarium of Pakistan, Museum of Natural History, Islamabad) and a voucher specimen (# 032564) of the plant was submitted. The study on plants complied with relevant institutional, national, and international guidelines and legislation.

### Ethics statement

No particular authorizations were required for the collection of plants for the described studies. Locations, where the plants were collected, were not in private-possession or secure in any way, and the field studies did not encompass endangered or protected species. Animals were euthanized using the CO2 inhalation method following ARAC guidelines. Animals were given favorable conditions (temperature 25^0^C, 12/12 h light and dark cycle, and humidity 60 ± 10% and pathogen-free environment). The rats were fed a dietary formulation of protein (18.1%), fat (7.1%), carbohydrate (59.3%), 125, and fiber (15.5%) with food and water being provided ad libitum. Studies reported in the manuscript were carried out in compliance with the ARRIVE guidelines.

### Extraction of plant material

Leaves of *M. royleanus* were shade dried and finely powdered (500 g), soaked with 2000 ml 95% of methanol twice for 48 h with occasional shaking, and then filtered. Under reduced pressure, the filtrate was dried (40 °C) giving a yield of 12.2%, then suspended in distilled water (50 ml) and fractions were prepared by adding subsequent solvents (200 ml twice) successively with an increase of polarity difference (n-hexane, ethyl acetate, chloroform, n-butanol) shake vigorously. These fractions were separated accordingly and the soluble residue was used as the aqueous fraction. Fractions were dried eventually with the yields to that of the methanol extract, n-hexane (7.5%), chloroform (3.4%), ethyl acetate (8.9%), *n*-butanol (7.8%), and residual aqueous fraction (10.7%)^32^.

### Sample preparation and LC/MS/MS analysis

Non-tumorogenic RWPE1 (CRL-11609), tumorogenic NB11 (CRL-2851) and NB26 (CRL-2852) cell lines were directly obtained from ATCC (Manassas, VA), whereas 22Rv1 (CRL-2505), LnCap (CRL-1740), C4-2 (CRL-3314), RWPE-1 (CRL-11609), PC3 (CRL-1435) and DU145 (HTB-81) cell lines were obtained. CWR22Rν1cells which had been treated with a vehicle or MEM (1 µm) for 24 h were used for the quantitative proteomics analysis followed by the repetition of the experiment three times to yield 6 replicates. Treated cells were then trypsinized, followed by centrifugation and washing with PBS to obtain cell pellets. These cell pellets were kept at − 80 °C for Nano LC/MS/MS analysis. School of Pharmacy Analytical Instrumentation Center Mass Spectrometry facility was utilized for protein sample preparation and Nano-LC/MS/MS. Proteins extraction was done from the frozen cell pellets after the addition of 0.3 ml ice-cold PBS by passing them through a 23-gauge needle 10–15 times. The resultant solution was then centrifuged at 10,000 g for 10 min at 4 °C to remove the cell lysates. Micro BCA (Thermo Fisher Scientific/Pierce) was used to determine the protein concentration of the extracts.

20 µg of protein sample was then digested overnight with the 1 µg of sequencing grade trypsin, followed by the sample preparation for the LC/MS/MS by the by C18 Zip-Tip purification according to the manufacturer’s protocol (Millipore Inc.). Before the LC/MS/MS, samples were suspended in water with 0.1% formic acid (v/v). The protocol of Li et al. ^33^ was performed after slight modification. 1 µg of the digest was analyzed by injecting over reverse phase BEH C18 column (100 μm × 100 mm), with 1.7 μm, 300 Å pore particles size (Waters Corp. Milford) using a Waters nano ACQUITY chromatography system. Elution of the peptides was performed by a column using a 180 min increasing organic gradient i-e Solvent A was water/0.1% formic acid (v/v), while solvent B was acetonitrile/0.1% formic acid (v/v). The gradient started at 3% B and increased with a linear gradient to 35% B at 130 min. At 140 min the gradient increased to 95% B and was held for 10 min and at 160 min the gradient returned to 3% to re-equilibrate the column for the next injection. Data-dependent MS/MS on a Q-Exactive Orbitrap mass spectrometer (Thermo Fisher Scientific Inc.) was used for peptide elution. The instrument resolution was set to 70,000, the AGC target was set to 106 counts, the scan range was from 300–2000 m/z, the MS scan was recorded in profile while the MS/MS was recorded in centroid mode, dynamic exclusion was set to 25 s.

### Data processing and protein identification by human database search

To search the data, LC/MS/MS acquisition techniques were followed against the Swiss-prot human proteome database along with the utilization of the Sequest HT internet searcher under the Proteome discoverer 1.4 programmings. An invalid disclosure cut off (less than 1%) was set for the recognition of proteins. Ensuring the identification of proteins, the LC/MS/MS information was adjusted utilizing the Chromalign programming software. Quantification of the peptides washed out somewhere in the range of 30 and 130 min was performed on the managed data utilizing SIEVE 21. At a set p-value of 0.05, a list of proteins was made for the proteins which were already identified. A threshold was applied to the data to filter out all identified proteins using a single peptide only (no one-hit wonders). Additionally, the data was filtered again to filter out peptides that have a coefficient of variance greater than 30% among 6 replicates.

### Pathway analysis

A list of differentially expressed proteins was compiled for the understanding of pathways modulated by MEM. Data from classification systems GO databases and PANTHER (Protein Analysis Through Evolutionary Relationships; http://www.pantherdb.org/) were used to classify these proteins based on their gene ontology (GO) descriptions. These proteins were predicted based on biological classes, protein classes, and molecular functions.

For the analysis of disease/function pathways, canonical pathways, and protein–protein interactions, Ingenuity Pathway Analysis Software (QIAGEN Inc., https://www.qiagenbioinformatics.com/products/ingenuity-pathway-analysis) was used. The projected results of protein–protein interaction networks and canonical pathways were produced by inputs of gene identifiers, log2 fold-changes, and p-values comparisons between the control and treated group.

### Cell viability assay

3-(4, 5-dimethylthiazol-2-yl)-2, 5-diphenyl tetrazoliumbromide protocol was performed to show the impact of MEM on the viability of C4-2, PC3, DU145, and CWR22Rν1 cell lines. The cells were plated (1 × 104 cells per well) in 1 ml of culture medium consisting of 10–200 µg/ml dilution of MEM in 24-well microtiter plates. Cells were kept in a humidified incubator for 24 h at 37 °C, 200 µl of 3-(4,5-dimethylthiazol-2-yl)-2,5-diphenyl tetrazoliumbromide (5 mg/ml PBS) was supplemented to each well and kept for two hours, 200 µl of DMSO were added to each plate which was then spun (1800 × g for 5 min at 4 °C). The readings at 540 nm wavelength were noted on a microplate reader. The impact of MEM on inhibition of growth was calculated as % cell viability as DMSO-treated cells were kept as control.

### Isolation of protein as well as immunoblotting

Antibodies against cdk2, cdk4, cdk6, cyclin B1, cyclin D1, cyclin D2, cyclin E, p27, p21, PSA, Poly (ADP-ribose) polymerase (PARP), Bcl-2, Bax, Bak, Bcl-XL, and AR were obtained from Cell Signaling Technology (Beverly, MA). Anti-mouse and anti-rabbit secondary antibody horseradish peroxidase conjugate were obtained from Amersham Pharmacia Life Sciences. The Bio-Rad DC Protein Assay Kit was purchased from Bio-Rad DC. Novex precast Tris–Glycine gels were obtained from Invitrogen. The annexin-V-FLUOS Staining Kit was purchased from Roche.

SDS-page and western blot analyses were performed as in a previously described protocol with slight modifications^34^. After treatment with MEM ice-cold lysis buffer was added to the cells (50 nmol/liter Tris–HCl, 150 mmol/liter NaCl, 1 mmol/liter EGTA, 1 mmol/liter EDTA, 20 MMOL/LITER NaF,100 mmol/liter Na3VO4, 0.5% Nonidet P-40, 1% Triton X-100, 1 mmol/liter PMSF, pH 7.4) with protease inhibitors (Calbiochem) incubated over ice for 20 min. For western blotting 12% polyacrylamide gels were used to resolve 40 µg of protein.

Transfer the membrane immediately in 5% non-fat milk buffer for 30 min for shaking. Prepare the anti-body solution in 3 ml of 5% non-fat milk. Prepare a plastic punch sealed at three sides. Transfer the membrane from the blocking buffer to the pouch with clean forceps. The membrane is kept in milk solution with the anti-body solution and seal overnight at 4 degrees and put on the shaker. Transfer the membrane in a box containing 1 × washing buffer for 2 min. Keep the box on the shaker at moderate speed. Transfer the membrane inbox containing 20 ml of 5% non-fat milk buffer. Pour 10 μl of secondary antibody in the 20 ml of milk buffer in which membrane is present. Let it shake for at least 2 h. Wash the membrane three times with 1 × washing buffer for 5 min. Prepare the ECL solution. Mix the solution from bottle A to B in a 1:1 ratio. Put the membrane on a flat surface covered with saran paper. Pour 4 ml of ECL solution on the membrane and spread it carefully all over the membrane. Keep the ECL solution on the membrane for at least 5 min, detected by chemiluminescence autoradiography.

### Gene expression analysis

RNA extraction and RT -PCR analyses were performed as in a previously described protocol with slight modifications^35^. Whole RNA was extracted (RNeasy Mini Kit (Cat No./ID: 74,104) from the cells using the following method. RNA dilution was determined by using a spectrophotometer at 260 NM and cDNA was prepared by following the manufacturer protocol (BioLabs E6300) using the kit. The reaction mixture was prepared to contain 10 µl FastStart Universal SYBR Green Master (Roche, Germany), 6 µM reverse primers, and 10 µg cDNA, with RNAase free water added to a total volume of 20 µl. The amplification and real-time analysis were done for 40 cycles with the following parameters; 95 °C (10 min) to activate of FastStart Taq DNA polymerase; 60 °C (1 min) for amplification and real-time analysis. The gene expression levels were determined using 2-ΔΔCT. The primer sequence used was;

TPD52 Sense 5′-GAGGAAGGAGAAGATGTTGC-3′,

TPD52 Antisense 5′-GCCGAATTCAAGACTTCTCC-3′,

GAPDH Sense 5′ AAGGTCGGAGTCAACGGATTTGGT-3.

GAPDH Antisense 5′-ACAAAGTGGTCGTTGAGGGCAATG-3′.

### Immunofluorescence microscopy

C4-2 and CWR22Rν1 cells were cultured in two-chamber glass slides as described previously. The previously described protocol was performed^35^. The following day, cells were treated with 40 µg/ml of MEM for 24 h. Once the chamber was removed, Phosphate buffer was used to rinse the slides, 2% paraformaldehyde was used to fix the cells and permeablized in methanol. Slides were rinsed with phosphate buffer and 2% serum was used as a blocking agent. For mounting DAPI from Invitrogen company was used applied and hematoxylin for counterstaining. The analysis was done by using a Bio-Rad Radiance system (2100 MP Rainbow) for imaging at the University of Wisconsin-Madison.

### In vivo tumor xenograft model

Athymic male mice were acquired from the San Diego Institute of NxGen Biosciences were kept under a contamination-free environment (12 h day/12 h night schedule), provided with a sterilized food ad libitum. CWR22Rν1 cells were selected for evaluating the in vivo impact of MEM as they generate fast tumors in mice. The implantation of CWR22Rν1 cells is also responsible for the secretion of marked quantities of PSA in the bloodstream of the mice. Cells were analyzed, suspended in complete RPMI medium 1640. Tumor xenografts CWR22Rν1 cells in mice were established by injecting cells (1 × 106) subcutaneously near tail mixed with RPMI plus (Collaborative Biomedical Products, Bedford, MA) Matrigel in a ratio of 1:1. Twelve mice were randomly selected into two groups containing six animals each. Mice first group served as control were fed with normal drinking water. Mice of groups two were injected with an intraperitoneal injection of MEM 2.5 mg. Throughout the study weight of the mice's body, food, and water used were noted two times a week. Tumor volume was calculated by the formula 0.5238 × L1 × L2 × H (L1 = long diameter, L2 = short diameter, and H = height of the tumor) and tumor sizes were measured twice weekly. Animals were euthanized using the CO2 inhalation method following ARAC guidelines when tumor volumes reached ~ 1200mm^3^. Blood samples were collected by the mandibular bleed and serum separated from the whole blood was stored at − 20 °C. Serum PSA levels were assayed by the PSA ELISA kit described above. H&E stained slides of lung, kidney, liver, heart, and brain were prepared to examine the possible toxic effects of MEM treatment.

### ELISA for PSA

To estimate levels of prostate-specific antigen in CWR22Rν1 C4-2 cells ELISA kit (Anogen, Ontario, Canada) was used following the manufacturer's protocol, using a technique of sandwich immunoassay to evaluate the level of PSA.

### Immunostaining

Tumor tissues were added to formalin (10%) and hematoxylin was used for staining and staining of eosin for morphological visualization. Sections were kept with specific primary antibody for 24 h incubation at concentrations of 1:75 for caspase 3 as well as histone 3-phosphate (cell signaling, MA) after that horse reddish peroxidase-conjugated secondary antibody was used, then diaminobenzidine/DAB (DAKO liquid DAB, CA) staining was done and counterstained with hematoxylin. All sections were cover with coverslips after mounting with paramount and analyzed by a qualified pathologist who was blind to the treatment group.

### Silencing of tumor protein D52 by SiRNA-TPD52

TPD52-siRNA (75 nM) was used to transfect and SiRNA scrambled (75 nM) purchased from Santa Cruz, Nucleofection Kit particular for CWR22Rν1 cells from Amaxa Biosystems (Gaithersburg, MD). Nucleofextor kit solution was used to suspend the cells according to the manufacturer’s protocol. Nucleofector solution (100 μl) was added to 2 × 106 cells along with SiRNA. Cells were then relocated to the vial present in the kit and nucleofection was done with an Amaxa Nucleofector apparatus. Cells were transfected using the T-001 pulsing protocol and were transferred into 100 mm plates containing 37 °C prewarmed culture medium. After transfection, cells were cultured and the medium was replaced with the fresh warm medium. Cells were treated with 40 μM MEM for 24 h and the protein lysates were prepared. Cell viability after transfection was also determined by following MTT assay. Using 2 μg of green fluorescent protein (GFP) we found 70–80% of transfection potential with this process.

### Cell invasion assay

Sixteen hours after the transfection of CWR22Rν1 cells, the medium was changed to fresh RPMI without FBS. One day later, 4 × 104 cells were subjected to a cell invasion assay using a commercial kit. RPMI supplemented with 10% FBS was used as a chemo-attractant. After 24 h, the number of invaded cells was evaluated by using a Multi detection system according to the manufacture’s protocol.

### Cell migration assay

We used a scratch wound healing assay of tissue culture monolayers to a 6 well plate format. By scratching the cell-substrate mechanically with a pin, we are capable to generate natural sized wounds in all wells of a 6 well plate. Imaging of the healing wounds with a microscope allows us to differentiate perturbations that affect cell migration. TPD52- siRNA transfected CWR22Rν1 cells were allowed to grow to full confluence in 6 well plates and were subsequently starved with RPMI containing 10% FBS medium. The cells were wounded with pipette tips by scratching in the center and washed with phosphate-buffered saline. Fresh prewarmed media at 37 oC was replaced. Images of the cells were taken after 24 h of incubation at 37 °C in a 95%: 5% (v/v) mixture of air and CO2. Experiments were performed in triplicate to determine the migration of transfected cells.

### Statistical analysis

The data obtained from LS-MS/MS from 6 controls and 6 treated samples were evaluated using software such as Chromalign, SIEVE 2.1, and IPA. The data obtained from qRT-PCR was evaluated using StepOne Software v2.2 RQ Study (Applied Biosystems/ Life Technologies Corp.) and was exported as RQmax and RQmin (2-∆∆Ct+/−∆∆Ct SD). These results represent the relative quantity of the gene of interest.

Whereas, RQmax and RQmin represent the incorporation of the standard deviation of the ∆∆CT into the fold change calculations. GraphPad Prism 5 software (GraphPad Software Inc.) was used for one-way analysis of variance (ANOVA) for the biological replicates of qRT-PCR data following Dunnett’s multiple comparison test.

### Ethics approval and consent to participate

This study makes use of mice and the experimental protocol for the use of mice was approved (BAS#0256) by the ethical board of the University of Wisconsin, Madison, USA.

### Consent for publication

Not applicable.

## Supplementary Information

Supplementary Information.

## Data Availability

All the data is contained in the manuscript.

## Abbreviations

MEM: Methanolic extract of *M. royleanus* leaves
PSA: Prostate-specific antigen
TRAMP: Transgenic adenocarcinoma of the mouse prostate
μg: Microgram
μl: Microliter
AR: Androgen receptor
BSA: Bovine serum albumin
IC50: Inhibition concentration fifty percent
PSA: Prostate-specific antigen
TPD52: Tumor protein D52
SiRNA: Small interfering RNA
