# Supplementary material for: Tissue microarray profiling and integrative proteomics indicate the modulatory potential of Maytenus royleanus in inhibition of overexpressed TPD52 in prostate cancers
Source: Sci Rep. 2021 Jun 7;11:11935. doi: 10.1038/s41598-021-91408-8 (PMC8184821; doi:10.1038/s41598-021-91408-8)
Supplement: Supplementary file 1 — Supplementary Information. [file 41598_2021_91408_MOESM1_ESM.pdf]

Supplementay File 1: IPA analysis

Tissue Microarray Profiling and Integrative Proteomics indicate the modulatory potential of *Maytenus royleanus* in inhibition of overexpressed TPD52 in prostate cancer

Maria Shabbir[1], Hasan Mukhtar[2], Deeba Syed[2], Suhail Razak[3*,] Tayyaba Afsar[3], Ali Almajwal[3], Yasmin Badshah[1], Dara Aldisi[3]

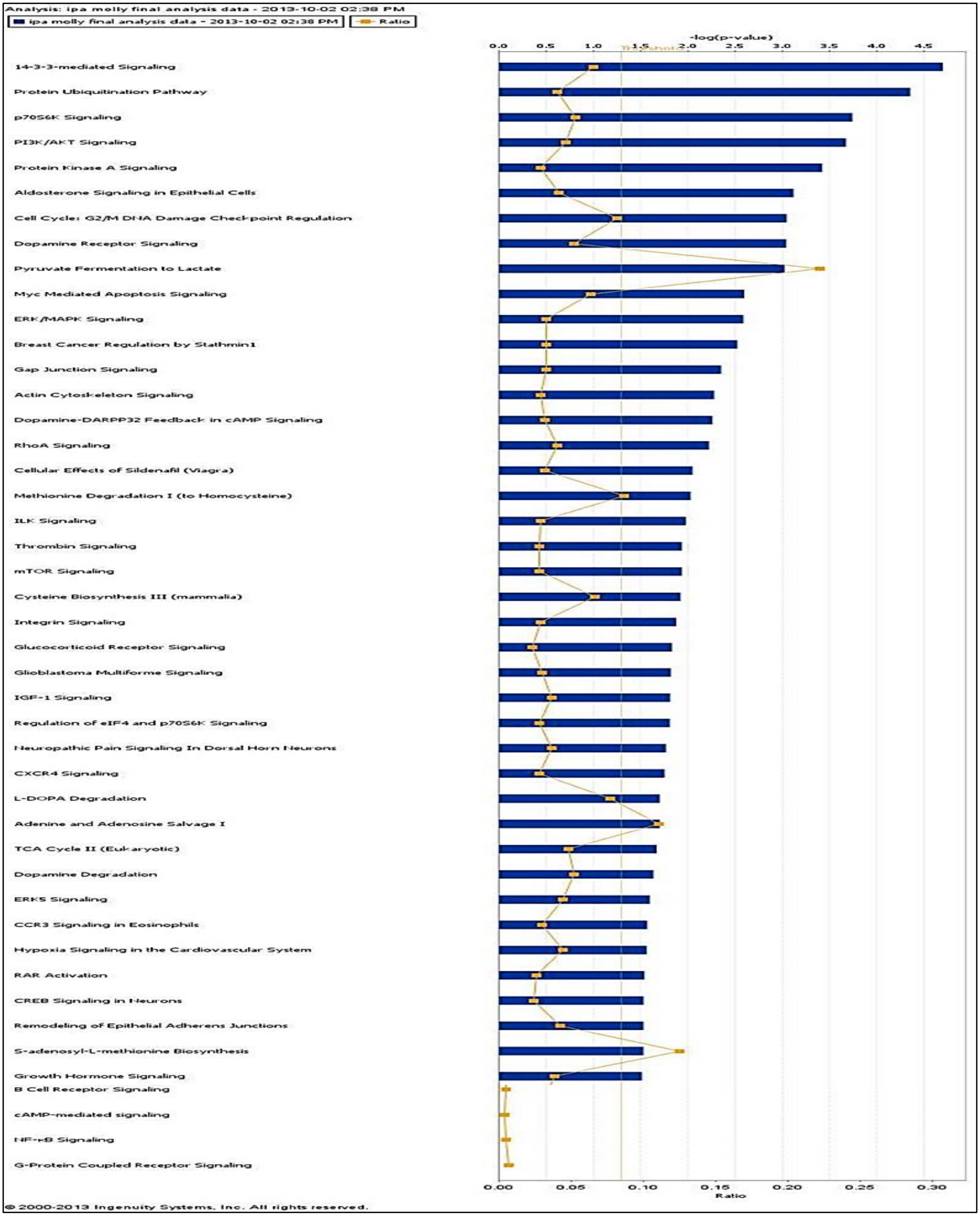

a) Association of canonical signaling pathways with modulated proteins are shown. The proteins which demonstrated significant change (95% confidence interval with statistical significance) were subjected to IPA analysis. The top 26 canonical pathways were identified as significantly altered upon MEM treatment. The line bar represents the threshold of significance (p = 0.05).

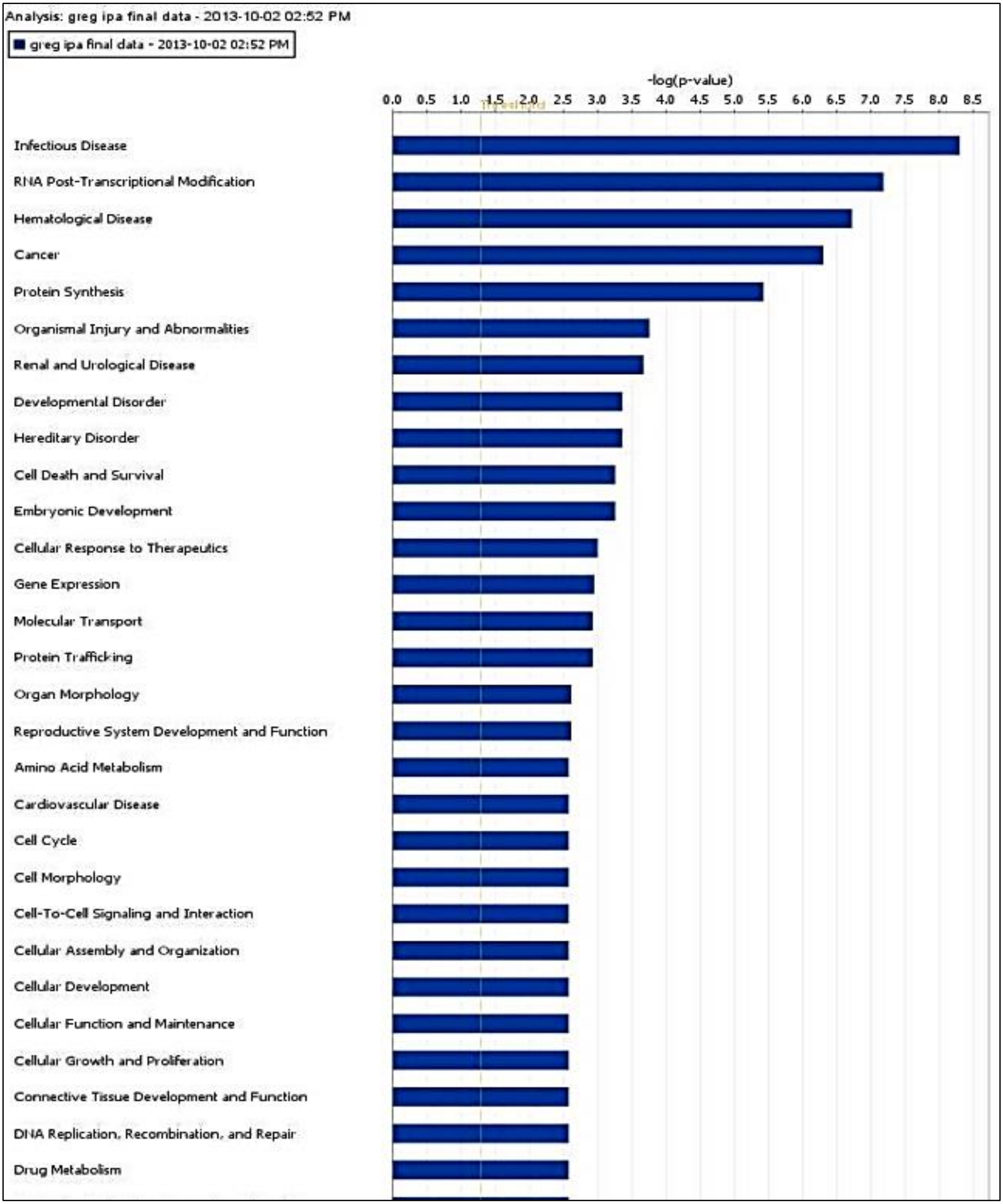

b) IPA was further used to categorize the proteins on the basis of disease and/or functional relation to the altered proteins.

Supplementary file S2: TPD52 in association with clinical PCa in a tissue microarray

Tissue Microarray Profiling and Integrative Proteomics indicate the modulatory potential of *Maytenus royleanus* in inhibition of overexpressed TPD52 in prostate cancer

Maria Shabbir[1], Hasan Mukhtar[2], Deeba Syed[2], Suhail Razak[3,] Tayyaba Afsar[3], Ali Almajwal[3], Yasmin Badshah[1], Dara Aldisi[3]

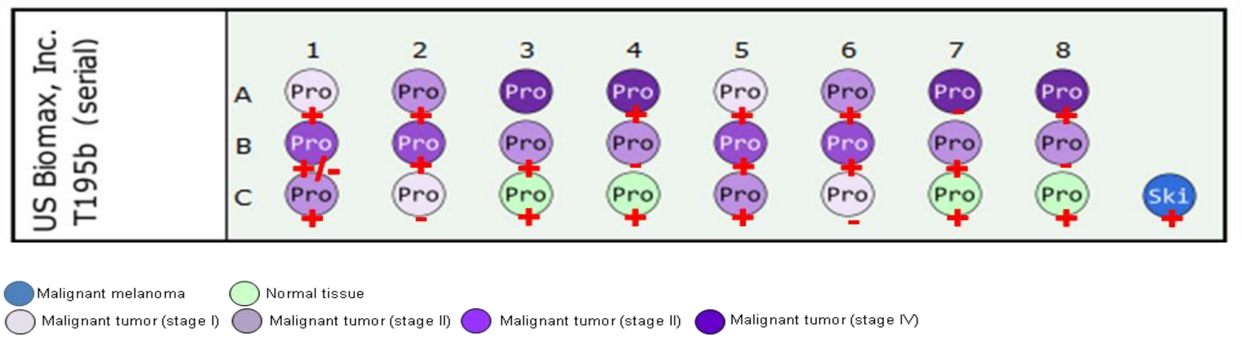

(a)Details of tissue microarray

Pos	No.	Sex	Age	Organ	Pathology diagnosis	Grade	Stage	Gleason Grade	Gleason Score	TNM	Type †
A1	1	M	64	Prostate	Adenocarcinoma	1	I	1	1+2	T1N0M0	Malignant
A2	2	M	73	Prostate	Adenocarcinoma	2	II	3	3+3	T2N0M0	Malignant
A3	3	M	73	Prostate	Adenocarcinoma	2-3	IV	4	4+4	T3N0M1	Malignant
A4	4	M	61	Prostate	Adenocarcinoma	1	IV	2	3+2	T3N1M0	Malignant
A5	5	M	64	Prostate	Adenocarcinoma	1	I	1	1+2	T1N0M0	Malignant
A6	6	M	73	Prostate	Adenocarcinoma	2	II	3	3+3	T2N0M0	Malignant
A7	7	M	73	Prostate	Adenocarcinoma	2-3	IV	4	4+4	T3N0M1	Malignant
A8	8	M	61	Prostate	Adenocarcinoma	1	IV	2	3+2	T3N1M0	Malignant
B1	9	M	70	Prostate	Adenocarcinoma	2	III	3	3+4	T3N0M0	Malignant
B2	10	M	66	Prostate	Adenocarcinoma	2-3	III	4	3+4	T3aN0M0	Malignant
B3	11	M	65	Prostate	Adenocarcinoma	1-2	II	2-3	2+3	T2N0M0	Malignant
B4	12	M	66	Prostate	Adenocarcinoma	3	II	5	5+5	T2N0M0	Malignant
B5	13	M	70	Prostate	Adenocarcinoma	2	III	3	3+4	T3N0M0	Malignant
B6	14	M	66	Prostate	Adenocarcinoma	2-3	III	4	3+4	T3aN0M0	Malignant
B7	15	M	65	Prostate	Adenocarcinoma	1-2	II	2-3	2+3	T2N0M0	Malignant
B8	16	M	66	Prostate	Adenocarcinoma	3	II	5	5+5	T2N0M0	Malignant
C1	17	M	62	Prostate	Adenocarcinoma	3	II	5	5+4	T2N0M0	Malignant
C2	18	M	69	Prostate	Low grade malignant leiomyosarcoma	-	Ia	-	-	T1N0M0 G1	Malignant
C3	19	M	33	Prostate	Normal prostate tissue	-	-	-	-	-	Normal
C4	20	M	43	Prostate	Normal prostate tissue	-	-	-	-	-	Normal
C5	21	M	62	Prostate	Adenocarcinoma	2	II	3	3+4	T2N0M0	Malignant
C6	22	M	69	Prostate	Low grade malignant leiomyosarcoma	-	Ia	-	-	T1N0M0 G1	Malignant
C7	23	M	33	Prostate	Normal prostate tissue	-	-	-	-	-	Normal
C8	24	M	43	Prostate	Normal prostate tissue	-	-	-	-	-	Normal
-	-	M	58	Skin	Malignant melanoma (tissue marker)	-					Malignant

b) Stages of malignancy of prostate cancer patients

Tissue Microarray Profiling and Integrative Proteomics indicate the modulatory potential of *Maytenus royleanus* in inhibition of overexpressed TPD52 in prostate cancer

Maria Shabbir[1], Hasan Mukhtar[2], Deeba Syed[2], Suhail Razak[3*,] Tayyaba Afsar[3], Ali Almajwal[3], Yasmin Badshah[1], Dara Aldisi[3]

Supplementary figure S3: Effect of MEM treatment on CWR22Rv1 tumor growth in athymic nude mice.

a)

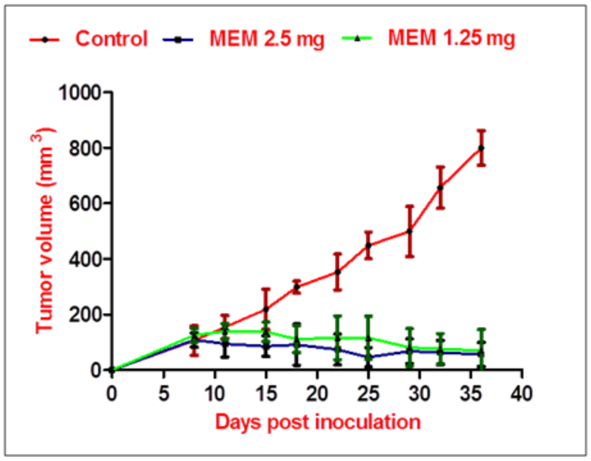

1)

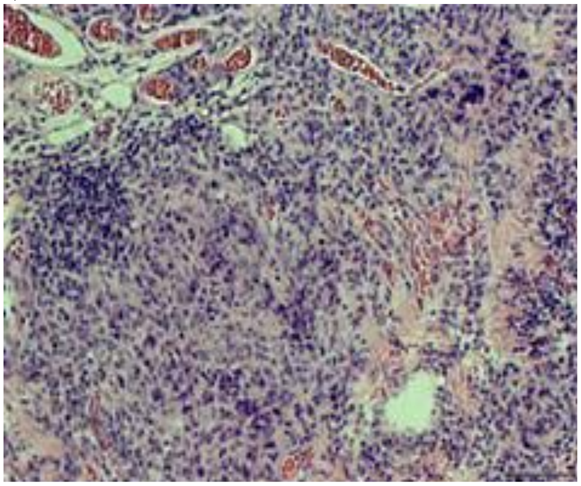

2)

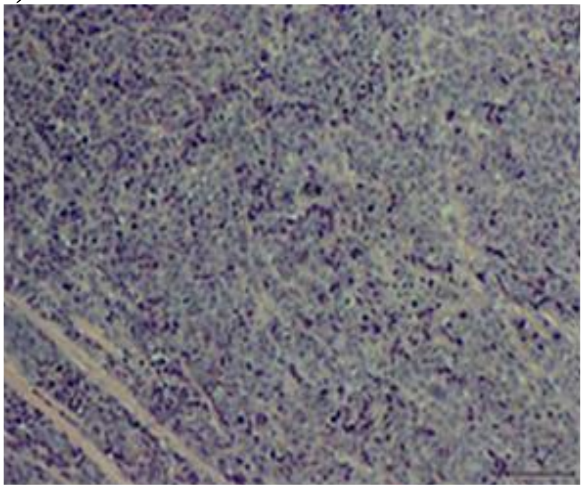

b)

Figure 1: Effect of Methanolic extract of *M. royleanus* leaves **(**MEM) treatment on CWR22Rv1 tumor growth in athymic nude mice. (a) Average tumor volume of water fed, 2.5 & 1.25 mg MEM

injected mice plotted over days after tumor cell inoculation. Values represent mean±SD of six mice. *, $p < 0.01$ (2.5 mg); **, $p < 0.05$ (1.25 mg) vs water fed normal control mice ***, $p < 0.001$. (b) H&E staining of MEM treated xenograft tumors (b1) vs control (b2).

S3: Effect of MEM treatment on tumor weight and various organs in athymic nude mice.

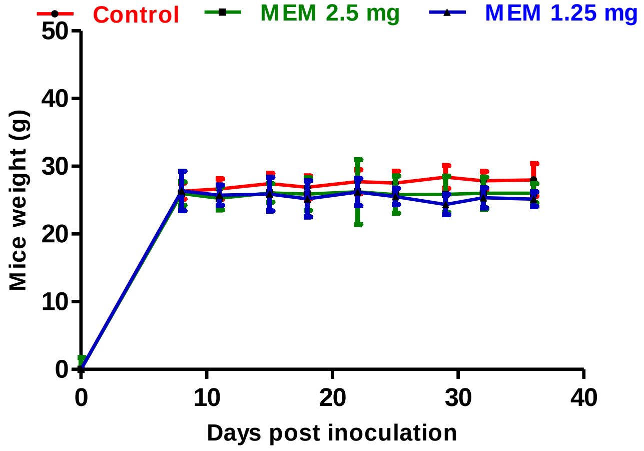

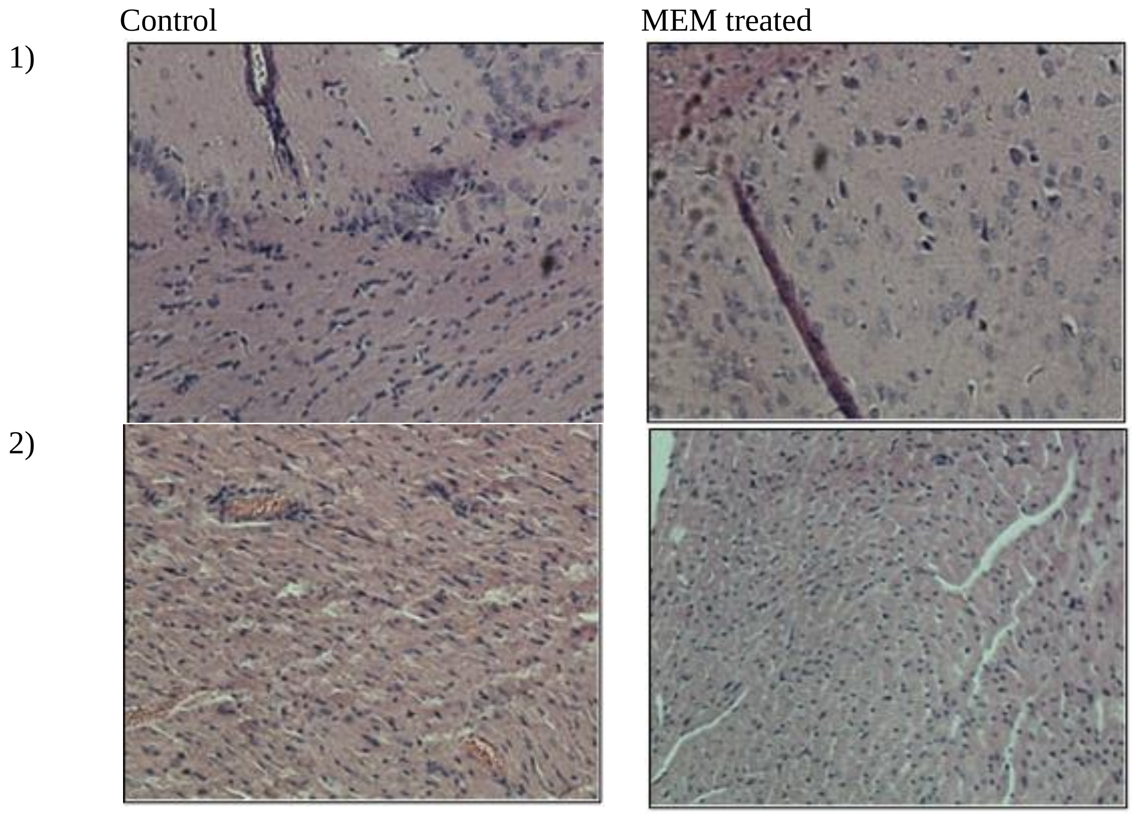

3)

4)

5)

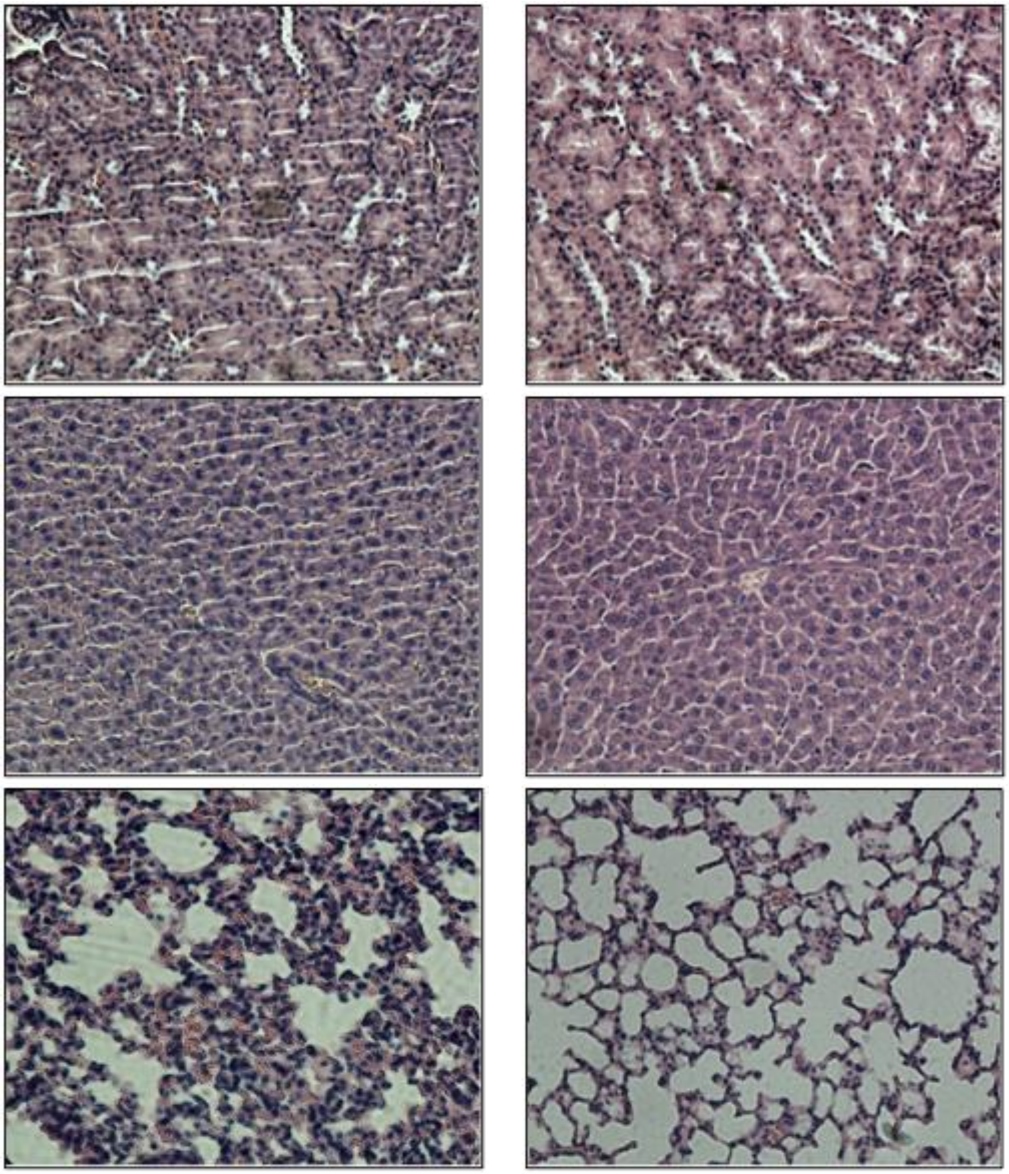

b)

Figure 2: Effect of methanol extract of *M. royleanus* leaves **(**MEM) treatment on tumor weight and various organs in athymic nude mice. (a) Mice weight was taken twice weekly and values represent mean±SD of six mice. **, $p < 0.02$ (2.5 mg),*, $p < 0.01$ (1.25 mg) vs control group;**, $p < 0.001$. (b) H&E staining of brain (1), heart (2), kidney (3), liver (4) and lung (5) of MEM treated mice with tumors vs. control (untreated tumors) for toxicity studies.

Supplementary S3Table 1: Report generated by pathologist

Ruth Sullivan, (Pathologist), VMD PhD, Diplomate ACVP

Slide review: Preliminary observations.

Organ	Control (athymic nude mice with tumor; untreated)	MEM treated athymic nude mice with tumor
**Liver**	1. Minimal randomly scattered mononuclear and supportive hepatitis<br>2. Minimal lymphoplasmacytic and histolytic portal hepatitis.<br>3. Sinusoidal brown pigment accumulation interpreted as probable artifact of red blood cell staining.	1. Capsular fibrosis and mild chronic mononuclear and mildly suppurative inflammation (suggestive of peritonitis).<br>2. Minimal mononuclear portal hepatitis.<br>3. Mildly enhanced hepatocellular mitotic rate, presumptive.<br>4. Locally extensive moderate accumulation of pigment laden macrophages/Kupffer cells<br>6. Minimal extra-medullary hematopoiesis
**<u>Brain</u>**	Extensive dark neuron artifact interpreted as an artifact of dissection. There is extremely rare mild extravasations of blood into Virchow-Robbins' space. The habenular nuclei have a mesh-work of cells (presumptive neurons) with smudged nuclear features.<br>Diagnoses:<br>1. Locally extensive nuclear smudging in the habenular nuclei (a finding of uncertain significance).<br>2. Minimal extravasations of blood into Virchow-Robbins' space.	There is fairly extensive dark neuron artifact (presumptive secondary to dissection).
**<u>Heart</u>**	There is rare individual cardiac myocytes with increased cytoplasmic eosinophilia and bland darkly staining contracted nuclei.<br>Diagnoses:<br>1. Minimal individual myocytes change, interpreted as probable degerative change.	There are rare individual cardiac myocytes with slightly more darkly eosinophilic cytoplasm than neighboring cells and with more homogenous and darkly eosinophilic chromatin staining in in contracted and shrunken nuclei.<br>Diagnoses:<br>1. Minimal individual myocytes change, interpreted as probable degerative change.

**<u>Kidney</u>**	No significant histological lesions are noted.	The renal capsule is segmentally broadened with fibrous connective tissue that is occasionally infiltrated with small numbers of mononuclear leukocytes and neutrophils.<br>Diagnoses:<br>1. Capsular fibrosis and mild chronic mononuclear and mildly suppurative inflammation (suggestive of peritonitis)
**<u>Lung</u>**	Neutrophils are moderately numerous percolating through alveolar septal walls and occasionally within alveolar air spaces. Occasional alveolar pneumocytes have expansive cytoplasm. The pulmonary parenchyma is multifocally collapsed, presumed secondary to dissection technique.<br>Diagnoses:<br>1. Pneumonitis, suppurative, moderate	The alveolar air spaces in some areas are mildly collapsed presumed secondary to dissection technique. There are rare megakaryocytes in the pulmonary parenchyma (EMH). There is scant alveolar hemorrhage presumed secondary to euthanasia. Neutrophils are in mildly enhanced numbers in alveolar air spaces and septal walls in a few scattered regions. The tip of the lung lobe has a focal accumulation of slightly increased numbers of foamy macrophages on alveolar septal walls and occasionally in alveoli. Uncommonly, pneumocytes lining alveolar septal walls have expanded cytoplasm (pneumocyte hypertrophy). There is one focus of perivascular lymphoid cuffing at the tip of one lung lobe.<br>Diagnoses:<br>1. Pneumonitis, mild, suppurative<br>2. Extramedullary hematopoiesis (EMH), mild

Additional file

Title: Tissue Microarray Profiling and Integrative Proteomics indicate the modulatory potential of *Maytenus royleanus* in inhibition of overexpressed TPD52 in prostate cancer

Maria Shabbir[1], Hasan Mukhtar[2], Deeba Syed[2], Suhail Razak[3,] Tayyaba Afsar[3], Ali Almajwal[3], Yasmin Badshah[1], Dara Aldisi[3]

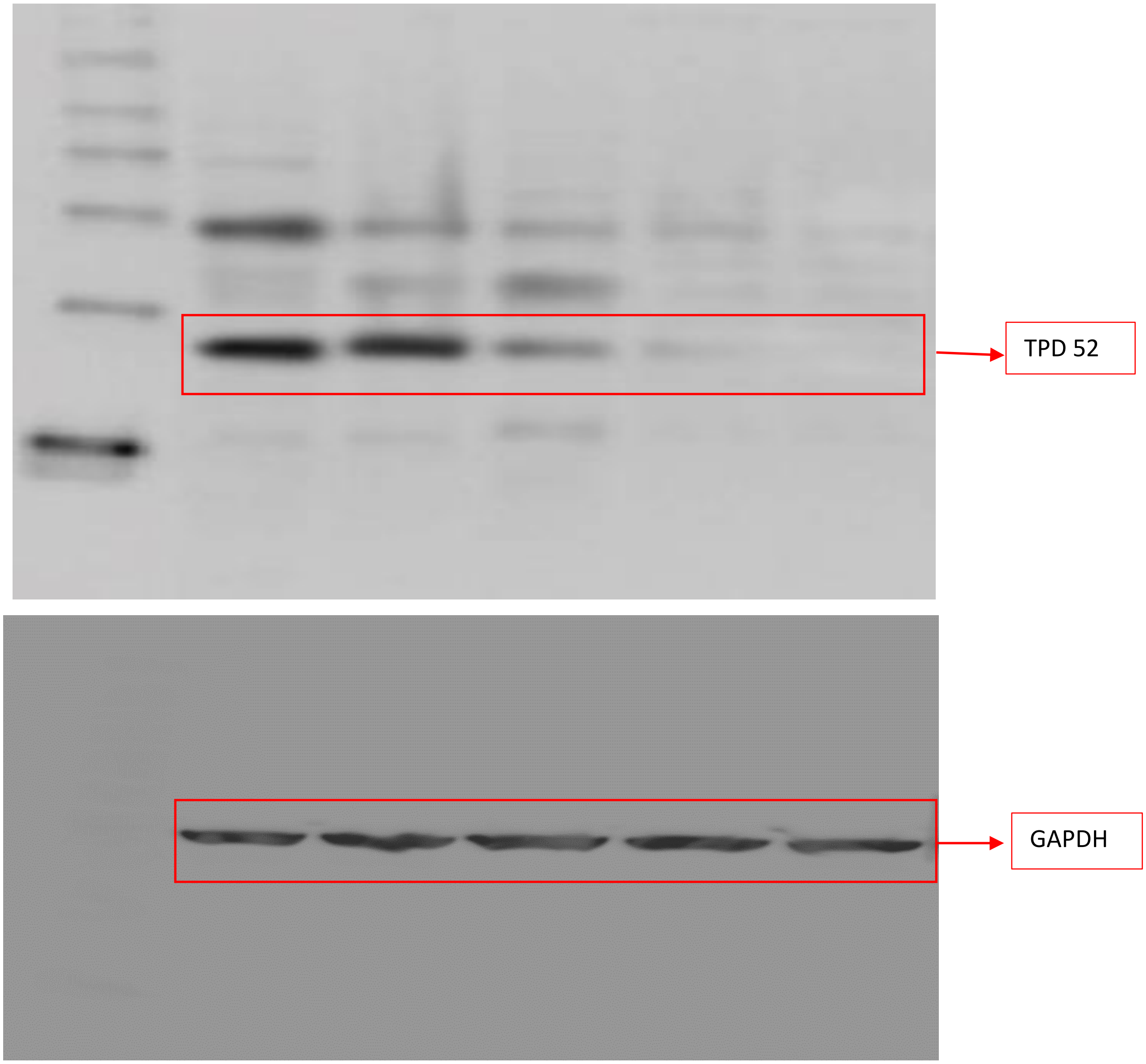

a) **Expression of TPD52 in Prostate cancer progression model**

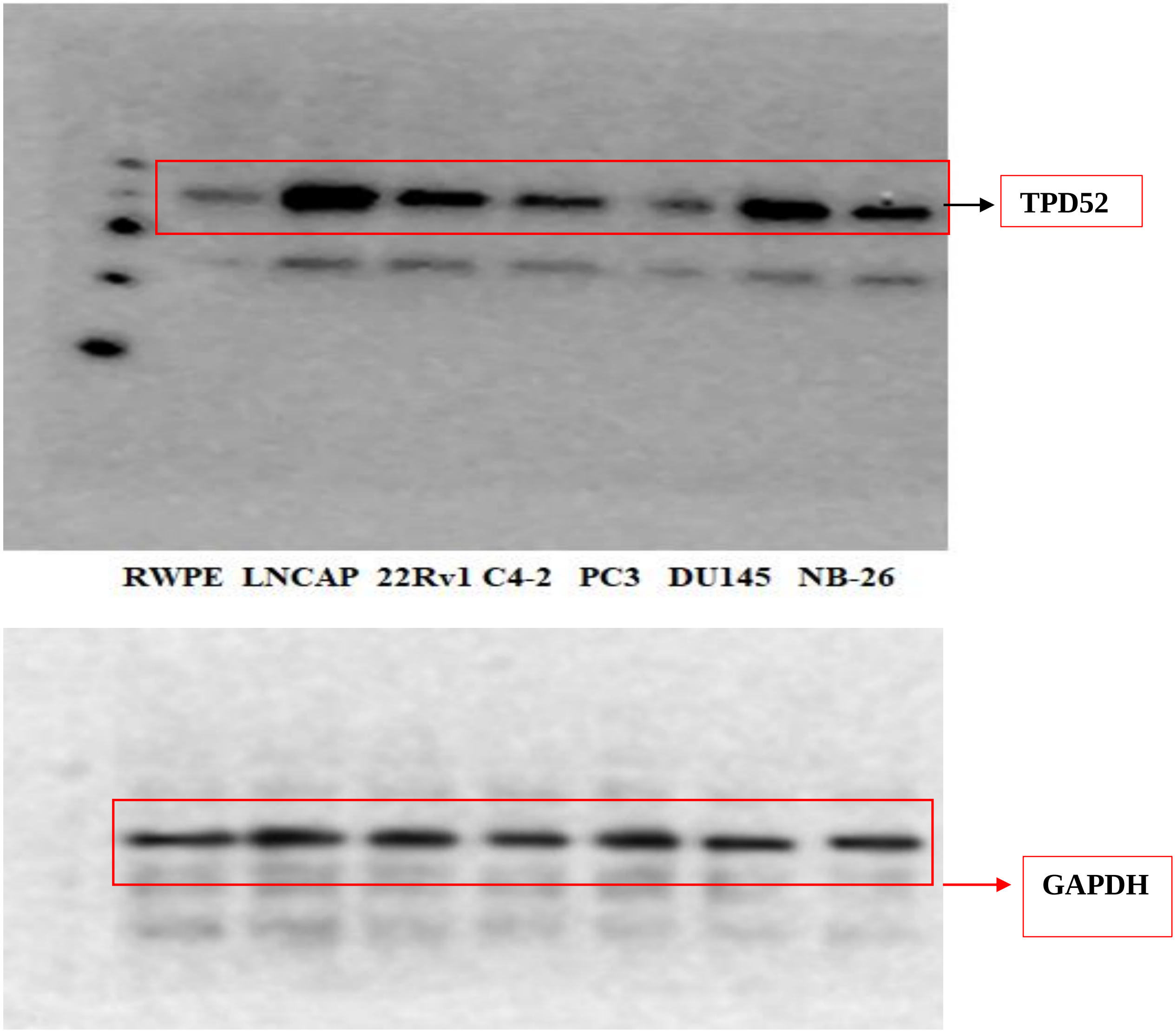

b) Expression of TPD52 in different prostate cancer cell lines

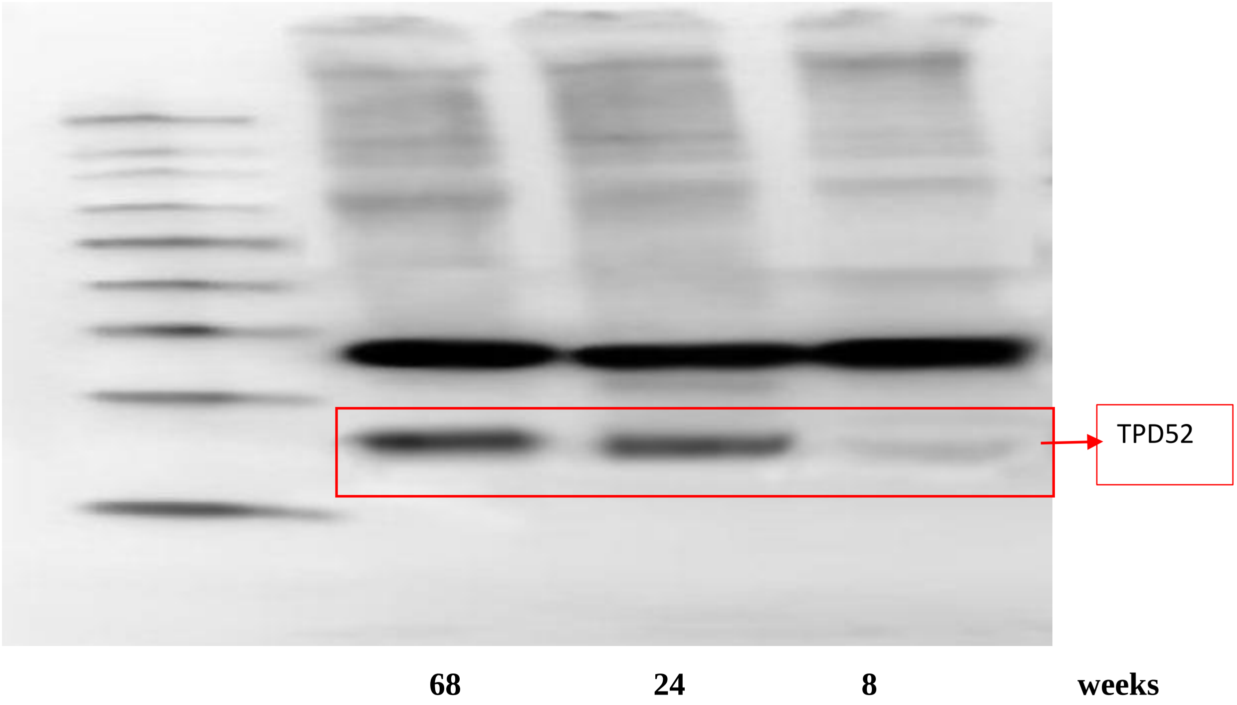

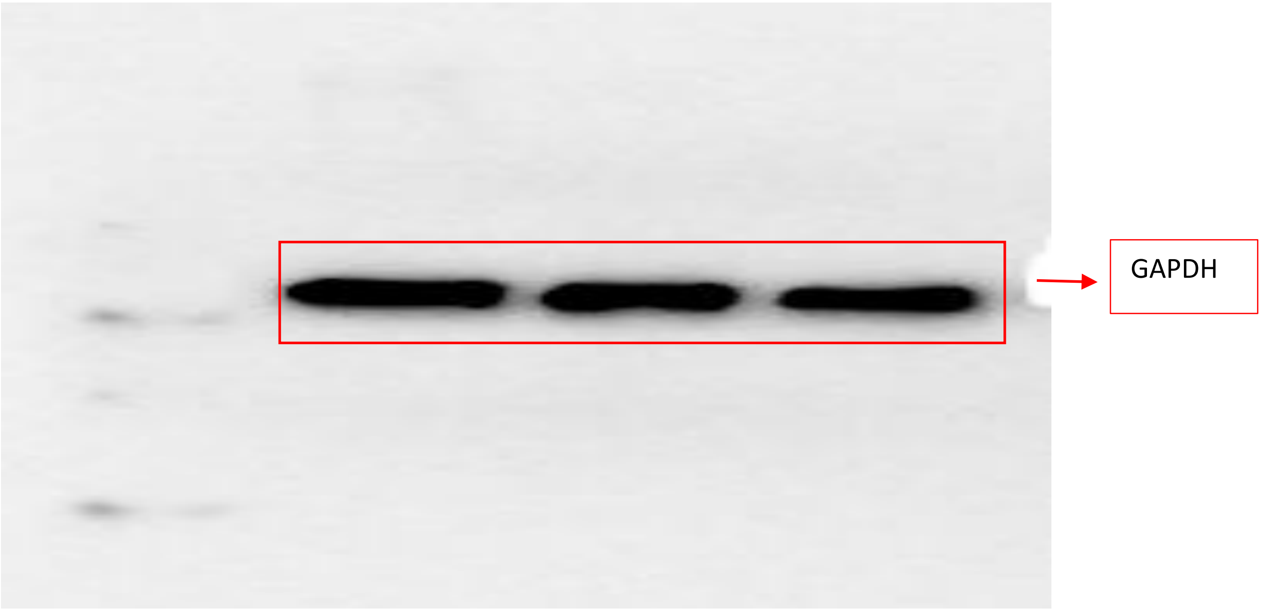

c) **Expression of TPD52 in different Prostate cancer progression and development**

Figure 4 : Immunoblot analysis for expression of TPD52 in prostate cancer development and progression model (transgenic adenocarcinoma of the mouse prostate, TRAMP), showed an increase in expression with development and progression of prostate cancer

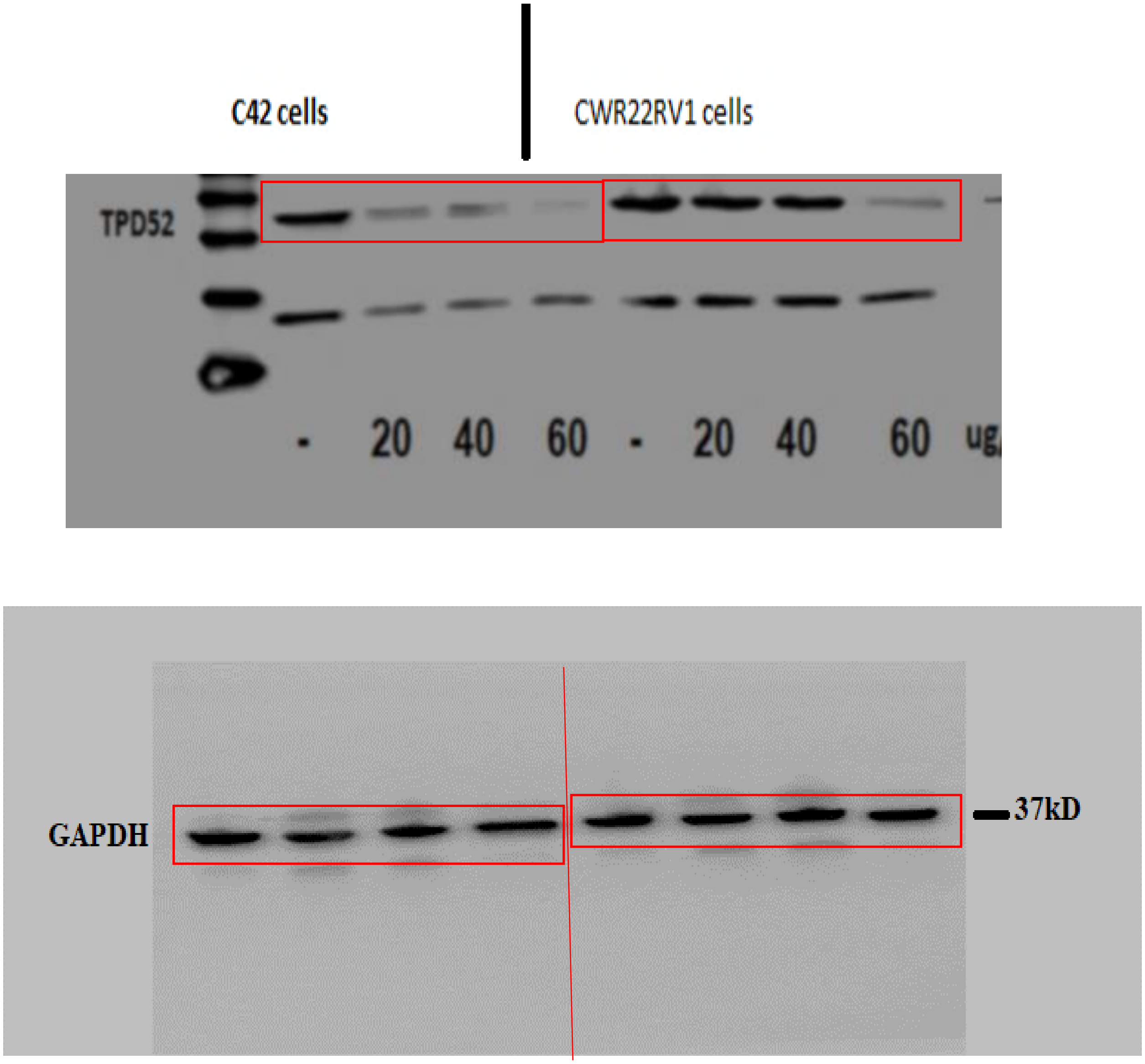

Figure 5: Modulation of TPD52 expression by MEM treatment in CWR22Rv1 (b1) and C_{4-2} (b2) cells. Equal loading of protein was confirmed by stripping the immunoblot and reprobing it for β-actin, experiment was done in triplicate.

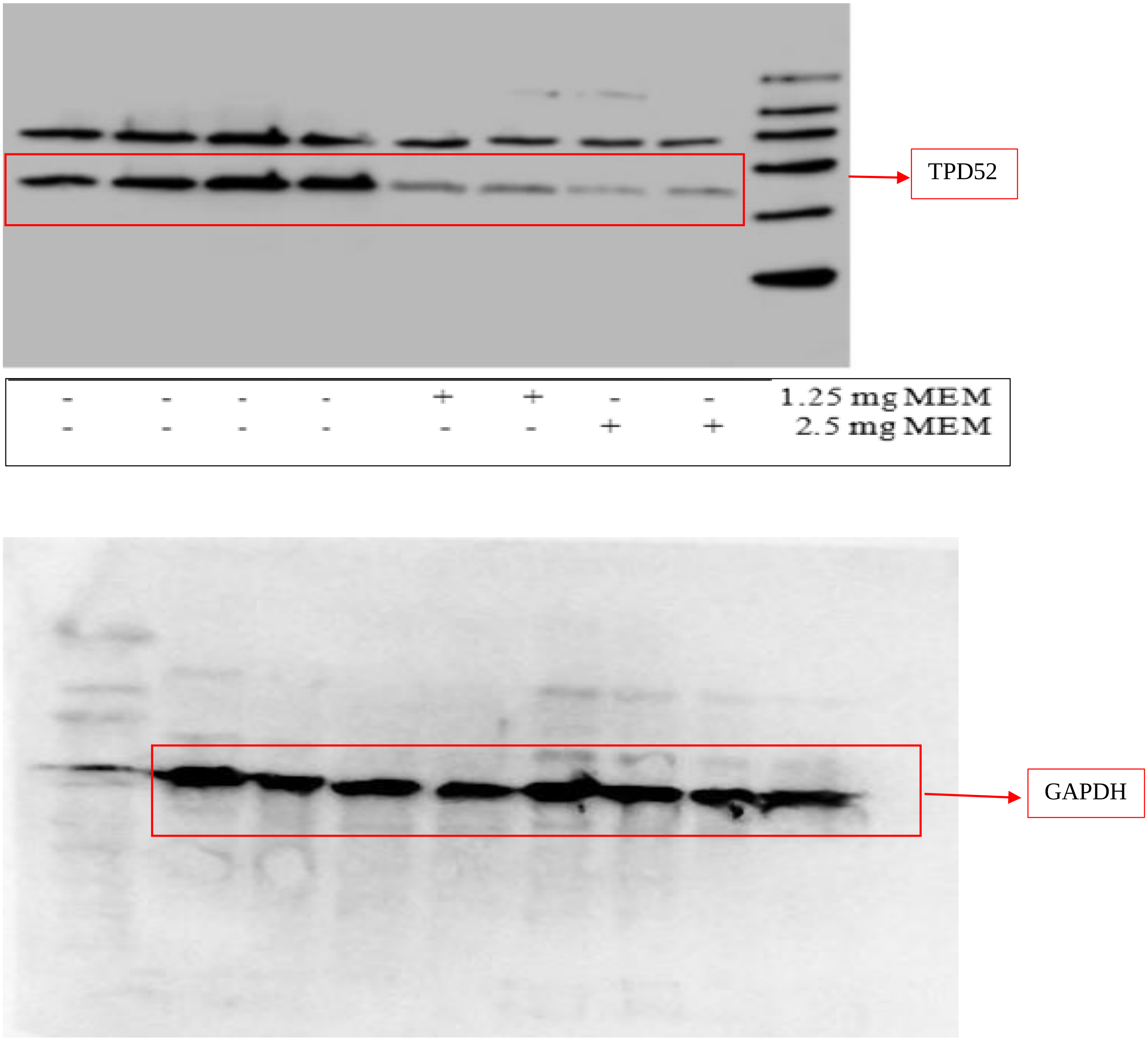

Figure 7: Validation of TPD52 expression in CWR22Rv1 xenografts tissues in athymic nude mice and effect of MEM treatment on TPD52 expression. a) Immunoblot analysis of TPD52 expression CWR22Rv1 xenografts tissues in MEM treated group as compared to control group. Total cell lysate was prepared and 40 µg protein was subjected to SDS-page followed by Immunoblot Analysis Blots shown here are representative of three independent experiments with similar results.

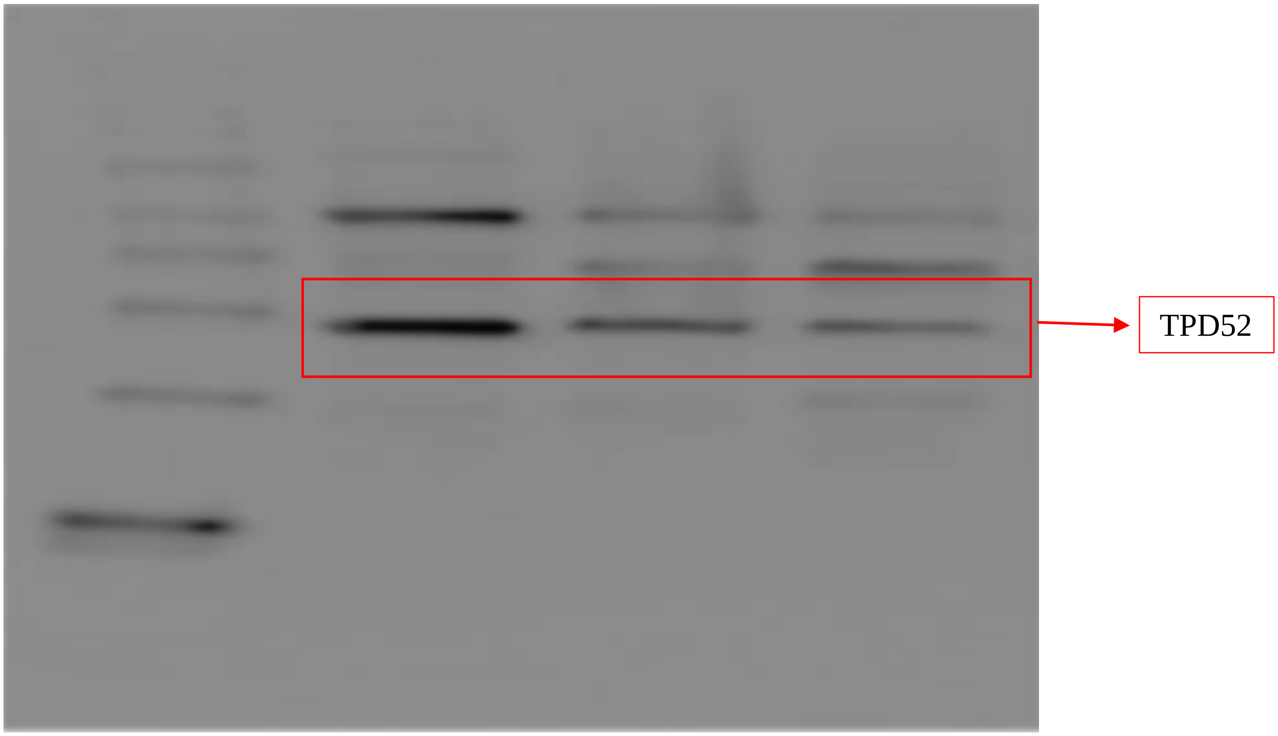
TPD52

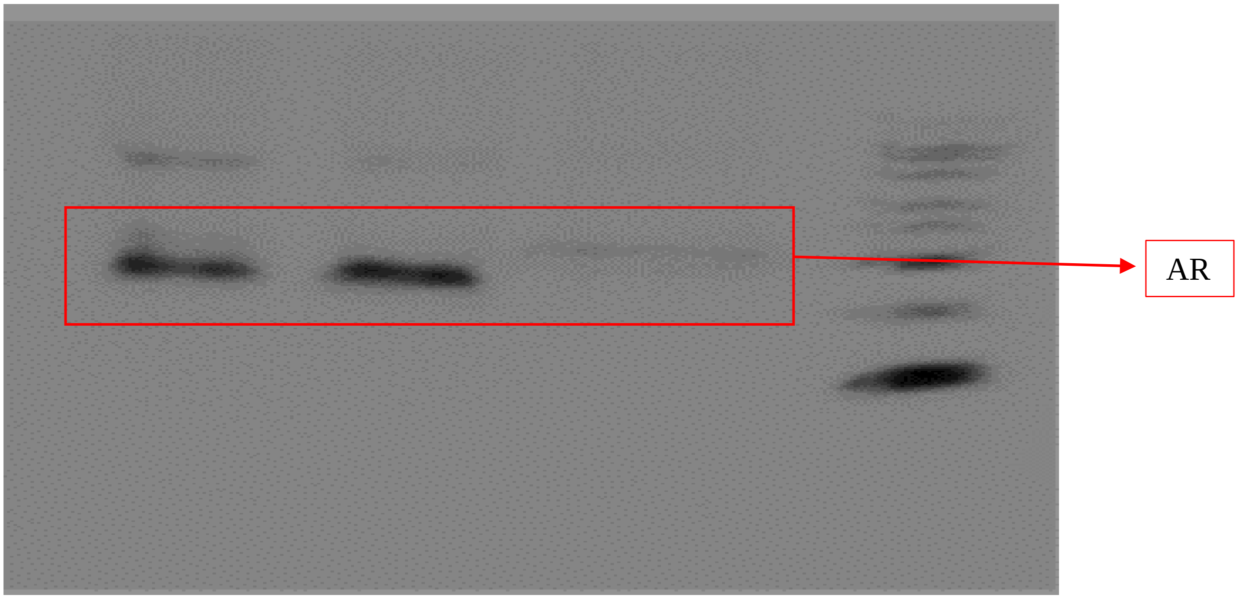
AR

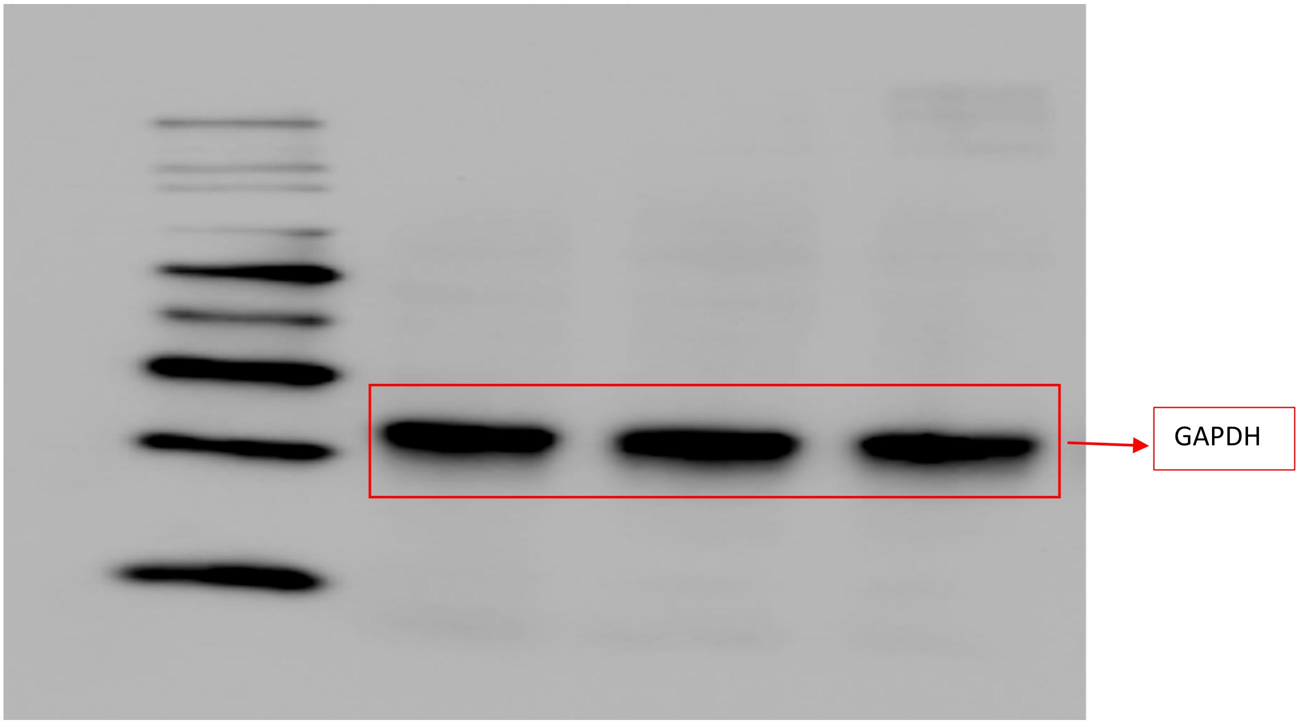

Figure 8: a) Western blot analysis of TPD52 protein expression in siRNA-TPD52 transfected CWR22Rv1 cells.
